# Analytical quaternion-based bias estimation algorithm for fast and accurate stationary gyro-compassing

**DOI:** 10.1038/s41598-024-66282-9

**Published:** 2024-07-09

**Authors:** H. Mohammadkarimi, S. Mozafari, M. H. Alizadeh

**Affiliations:** Department of Aerospace Engineering, Amirkabir University of Technology, Tehran, Iran

**Keywords:** Gyrocompassing, Azimuth alignment, Initial alignment, Strapdown INS, Inertial sensor errors, Kalman filter, Astronomy and planetary science, Energy science and technology, Engineering, Mathematics and computing, Physics

## Abstract

This work introduces a novel approach to Strapdown Inertial Navigation System (SINS) alignment, distinct from recursive methods like Kalman filtering. The proposed methodology expedites bias error calculations by utilizing quaternion-based analytical relationships, which bypasses the slow convergence behavior associated with recursive algorithms, particularly in azimuth angle error estimation. In addition, the proposed approach demonstrates comparable accuracy to traditional fine alignment methods. Simulations and experiments validate that in contrast to the 10-min time requirement of traditional fine alignment methods (for azimuth angle estimation in stationary conditions), the proposed approach achieves the same accuracy within 20 s. However, limitations exist as the algorithm is applicable only in stationary conditions, and necessitating a high-grade IMU capable of measuring the earth’s rotation rate.

## Introduction

A strap-down inertial navigation system (SINS) operates based on solving inertial navigation equations, relying on data from accelerometers and angular rate sensors attached to the vehicle as a package named Inertial Measurement Unit (IMU)^1^. Errors in SINS solutions stem from three primary sources including initial conditions errors, sensor errors and navigation algorithm errors^2^. Alignment techniques aim to refine the accuracy of SINS solutions by mitigating or minimizing errors originating from the initial condition and sensor errors. The initial attitude error notably influences the positional accuracy of SINS. Specifically, errors in initial tilt (roll and pitch) exert a quadratic influence on position error over short durations, while errors in initial heading exhibit a cubic impact over the same timeframe^2^. Consequently, there is a substantial demand within the navigation community for alignment algorithms capable of accurately estimating initial attitude errors and sensor biases, given their significant impact on positional precision.

Initial alignment can be accomplished by processing data from the IMU solely^3^ or with the aid of supplementary sensors such as Global Positioning System (GPS)^4,5^, doppler velocity log^6,7^, camera^8,9^, odometer^10^ or the low-orbit satellite data^11^. However, In strategic applications such as satellite launch vehicles (VLS), it is crucial to perform alignment without any external assistance^12^ and this study proposes a new initial alignment algorithm utilizing solely IMU data, commonly referred to as self-alignment.

Navigation accuracy in SINS predominantly relies on the initial azimuth’s precision. Hence, azimuth alignment is a crucial issue in navigation^1,2,13–16^. The typical initial alignment procedure involves a two-step self-alignment process, including coarse and fine alignment^14^. The primary objective of the coarse alignment is to serve as an initial reference for the fine alignment process by providing approximate estimations of the misalignment angles within a narrow range of a few degrees^17,18^.

The coarse alignment can be categorized into two primary methodologies, distinguished by their reliance on unique observation vectors. One approach utilizes apparent gravity to determine the initial attitude of SINS, while the other is based on apparent velocity^19^. In recent years, there has been a growing interest in optimization-based alignment methods. These approaches link the SINS alignment and attitude determination problems using infinite vector observations over a continuous time interval. For instance, in^20^, the q-method, commonly employed to address the attitude determination problem, is used to heuristically transform the SINS alignment problem to an optimization problem of finding the minimum eigenvector. However, it cannot estimate the sensor biases and lacks a dependable quality indicator for evaluating the accuracy of OBA’s attitude. The need for a reliable quality index has been thoroughly investigated in^21^, where a real-time attitude accuracy indicator for the subsequent fine alignment is introduced through the covariance analysis of the OBA approach.

Although gyroscope biases are the most critical factors that affect the azimuth alignment accuracy of the INSs^22^, there exist limited approaches that take into account the Inertial IMU’s bias error in coarse alignment stage^23^. The approach presented in^24^ studies the estimation of biases and introduces an innovative coarse bias estimation method by employing orthonormality constraints for precise bias estimation. Although the algorithm has significantly reduced in-field bias estimation time, in applications requiring extremely high accuracy, this algorithm is not a suitable choice to perform the whole initial alignment procedure, as it doesn’t eliminate the need for a fine alignment stage. The approach outlined in^25^ determined the gyroscope bias independently of the accelerometer bias by incorporating a recursive Bayesian filter known as MUSQUE. However, accuracy will be compromised with a single iteration of the recursive filter and several iterations will be necessary. Ref.^22^ proposes a gyroscope multi-position bias estimation method that relies on attitude-tracking information and addresses the ill conditioned problems in gyroscope observation equations across three orientations. However, its practicality is constrained as the procedure necessitates orienting the IMU in at least three distinct positions, which restricts the method from applying to stationary vehicles.

The primary objective of the fine alignment procedure is to enhance the initial attitude estimation of the vehicle while also estimating any uncompensated biases in the inertial sensors (referred to as calibration)^26^. The fine alignment problem has drawn the interest of researchers globally, and the exploration for more rapid stationary alignment and bias estimation algorithms has been thoroughly documented in the existing literature. Literature^27^ introduces a strategy centered on expanding measurements to achieve a more rapid and accurate estimation of sensor biases. Despite its effectiveness, this approach does not enhance the convergence rate of platform misalignments, as it relies on unobservable uncompensated biases beyond the Kalman filter’s estimation capability. In^28^, a novel method is presented for initial alignment, utilizing a Kalman filter for fine alignment and incorporating horizontal gravity acceleration into measurement equations to improve attitude angle tracking. Meanwhile^29^, proposes a multi-rate self-alignment algorithm to boost the Kalman filter’s bias estimation capability. Ref.^30^ suggests a design principle for a dual-axis rotating SINS to address slow convergence and low accuracy during initial alignment and self-calibration. Conventional fine alignment algorithms exhibit divergence in cases where the initial azimuth angle error is significant. The main reason for such behavior is their inherent strong nonlinearity and limited observability, as outlined in prior works^31,32^. In recent years, Novel approaches using the theory of the Lie group have been introduced to immune the fine alignment algorithm to the initial large misalignment. These methods employ different types of Kalman filtering, such as the linear Kalman filter^33^, the invariant Kalman filter^34,35^, and the state transformation extended Kalman filter^36^, to improve convergence speed and enhance overall stability.

All fine alignment methods use recursive estimation algorithms such as the Kalman filter to adjust the velocity, attitude, and sometimes position based on the difference between the INS outputs and a reference^26^. Ref.^15,16^ have performed observability analysis of the attitude error model with velocity measurement. They have proved that the azimuth angle error is not fully observable, requiring a substantial duration for the filter to estimate it accurately^37^. Hence, the recursive filtering method has the drawback of a slow convergence speed for the azimuth angle.

The literature suggests that all coarse alignment algorithms demonstrate swift convergence; however, their drawback lies in their inadequate accuracy for initializing a SINS algorithm. Conversely, fine alignment procedures typically necessitate 10 min to converge to an acceptable azimuth angle calculation under stationary conditions^24^. Consequently, there is a need for a seamless technique in SINS alignment that combines the rapidity of coarse alignment with the accuracy of fine alignment methods.

This paper introduces a novel stationary SINS alignment procedure that precisely determines the azimuth angle within 20 s. Consequently, this study makes two significant contributions: 1- The proposed SINS alignment technique eliminates the need for recursive estimation algorithms, such as Kalman-based filtering methods, which can be hindered by a slow convergence rate when estimating azimuth angle error. Instead, analytical quaternion-based relationships were employed to determine bias errors, providing a significantly faster alignment duration; and 2- The algorithm calculates inertial sensor biases with very high accuracy and thus the bias compensation procedure is performed excellently.

Bias errors often degrade the inertial sensor’s quality and can significantly affect SINS performance; By accurately determining them, the introduced method allows for precise calculation of azimuth angle in SINS. Furthermore, over time, inertial sensors tend to experience bias-instability^38^. Therefore, developing a fast bias estimation technique presents an opportunity to disregard the bias-instability tendencies of inertial sensors, resulting in an expedited alignment process.

The accuracy of the proposed method in estimating azimuth angle and gyroscope biases is the same as the maximum accuracy achievable in traditional fine alignments. In addition, the algorithm’s execution time was investigated through numerical simulation in 200 different conditions. Finally, the analytical bias estimation relationships were validated experimentally using an FOG-based IMU in 50 different stationary conditions. As a result, the algorithm presents an alternative to conventional approaches for initial alignment in SINS equipped with high-grade IMUs.

The rest of this paper is organized as follows: Sect. "Proposed method" introduces the proposed method for the initial alignment of SINS as an algorithm. In Sect. "Numerical simulation and experimental results", the proposed method’s time performance and precision are verified through simulation and experiment. Finally, in Sect. "Conclusion", the advantages and disadvantages of the suggested method are discussed in detail.

## Proposed method

The purpose of this section is to present a comprehensive explanation of a new algorithm designed to align SINS systems quickly and precisely. Conventional alignment processes involve two main stages: leveling and north-finding^26^. In the proposed algorithm, the leveling Euler angles are assumed to be provided by a triad of accelerometers. Hence, the proposed algorithm focuses solely on determining the geographical north.

It can be demonstrated that the accuracy of the north-finding algorithm is closely tied to the accuracy of the inertial block. In theory, if the error of the inertial block is eliminated (ideal sensor), the error of the alignment algorithm will also approach zero. Hence, the algorithm’s accuracy can be enhanced by estimating and compensating for sensor errors.

**Figure 1:**
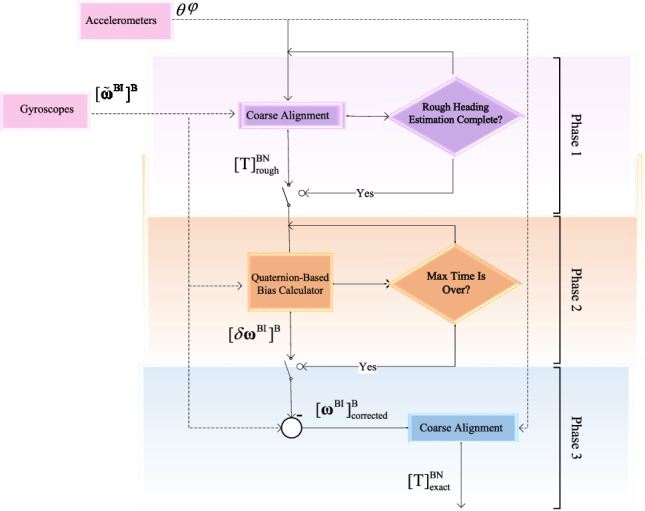
General view of the quaternion-based Differential Alignment process.

The proposed algorithm improves the traditional methods by using analytical relations to find and correct the errors of the gyroscopes. Instead of using a slow and complex fine alignment stage like other optimal-filtering-based algorithms, the outlined methodology calculates the initial attitude Euler angles in three phases. This process involves two simple coarse alignment phases and one error correction phase. This way, the algorithm can achieve high accuracy in a shorter time. The algorithm combines the advantages of both fast but inaccurate coarse alignment methods and slow but accurate fine alignment methods. The authors call this new algorithm “Quaternion-Based Differential Alignment” abbreviated as QBDA and show how it works in Fig. 1. In this figure, $\varphi$ and $\theta$ denote the roll and pitch Euler angles. ${\left[ {{{{\varvec{\tilde{\omega }}}}^{{\textrm{BI}}}}} \right] ^{\textrm{B}}}$ represents the measurements from the gyroscopes, while ${\left[ {\delta {{\varvec{\omega }}^{{\textrm{BI}}}}} \right] ^{\textrm{B}}}$ signifies the gyroscopes’ bias errors. $\left[ {{{\varvec{\omega }}^{{\textrm{BI}}}}} \right] _{\textrm{corrected}}^{\textrm{B}}$ corresponds to the corrected gyroscope measurements. Additionally, $\left[ {\textrm{T}} \right] _{\textrm{rough}}^{{\textrm{BN}}}$ indicates the coarse alignment output, which provides a rough estimate of the transformation matrix from the body frame to the navigation frame. Finally, $\left[ {\textrm{T}} \right] _{\textrm{exact}}^{{\textrm{BN}}}$ denotes the ultimate output of the proposed method, offering an exact estimation of the initial INS attitude represented by a body to navigation frame transformation matrix.

Figure 1 demonstrates that the proposed algorithm is divided into three phases. The second phase repeats itself until it reaches the maximum time limit of $t_{max}$, but the first and third phases only happen once. The first and third phases utilize a conventional coarse alignment algorithm, which is not covered in this article but can be found in^26^. It is essential to emphasize that the proposed algorithm relies on initializing the heading through a traditional direct gyrocompassing method. Consequently, the algorithm’s functionality is limited to high-grade IMUs. Hence, The IMU which is used in the gyrocompass algorithm should be as accurate to measure the Earth’s rotation rate. On the other hand, the second phase of Fig. 1 is the main focus of this research and is responsible for calculating bias errors in gyroscopes using analytical relations. In other words, the proposed algorithm refines the gyroscope sensors’ outputs by estimating their biases and calculates the north direction using a conventional coarse alignment algorithm. Hence, it can eliminate the need for time-consuming fine alignment techniques by employing analytical relations. Algorithm 1 represents all phases and their execution sequence in more detail.

**Algorithm 1:**
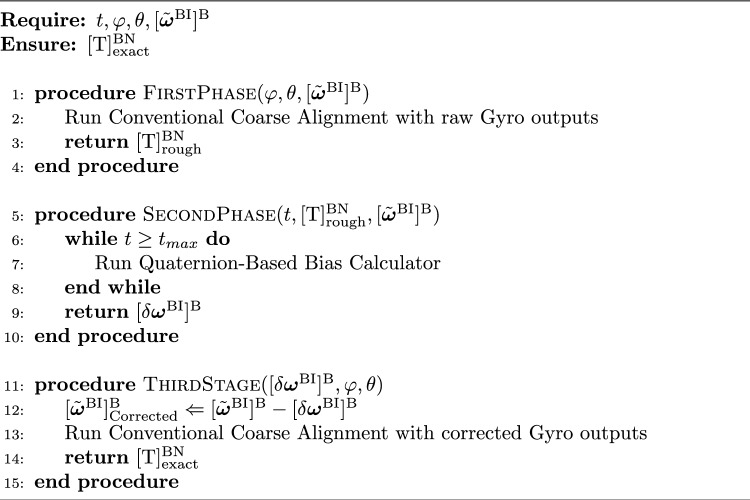
North finding algorithm

According to Algorithm 1, “first phase” receives tilt angles ($\varphi ,\theta$) and raw gyro data $[\tilde{\varvec{\omega }}^{\text {BI}}]^{\text {B}}$ as inputs and runs a conventional coarse alignment algorithm to return navigation to body frame transformation matrix ($[\textrm{T}]^{\text {BN}}_{\textrm{rough}}$) as a rough estimation of INS attitude.

Phase two starts with receiving current time (*t*), Initial guess ($[\textrm{T}]^{\text {BN}}_{\textrm{rough}}$) and gyro raw data ($[\tilde{\varvec{\omega }}^{\text {BI}}]^{\text {B}}$). After that, gyro biases ($[\delta \varvec{\omega }^{\text {BI}}]^{\text {B}}$) are estimated through a series of analytical equations inside an iterative loop. The user sets the maximum time of the iteration. Phase two contents are discussed deeply in Sect. "Second phase".

The third phase starts when the second phase runs out of time ($t_{\textrm{max}}$). It removes the biases ($[\delta \varvec{\omega }^{\text {BI}}]^{\text {B}}$) that were calculated from the gyro raw outputs following that does a standard coarse alignment, similar to the phase one. Phase three output is the exact INS attitude estimate shown by the navigation to body transformation matrix ($[\textrm{T}]^{\text {BN}}_{\textrm{exact}}$).

### Second phase

**Figure 2:**
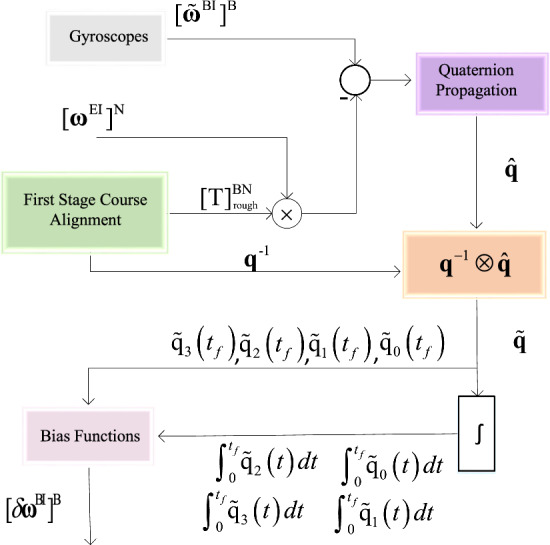
Second phase detail view.

This section offers an in-depth explanation of the “second phase” procedure as outlined in Algorithm 1. It also encompasses the derivation of the analytical equations employed within this procedure. In Fig. 2, you can observe the various components of the second stage procedure, which include the attitude propagation block, the attitude error calculation block, a summation block for the integration of attitude quaternion errors, and the primary contribution of this research - the bias functions block. The bias functions block is crucial in extracting inertial sensor bias errors using quaternion-based analytical equations. Subsequent subsections will introduce these bias calculator functions and provide an extensive description of each block.

#### Quaternion propagation

Quaternion propagation block in Fig. 2 propagates attitude one step forward. Consider the quadrature vector of quaternions as follows:

1 $$\begin{aligned} {\textbf{q}}^{\text {BN}} = \begin{bmatrix} {{q}_{0}} \\ {{q}_{1}} \\ {{q}_{2}} \\ {{q}_{3}} \\ \end{bmatrix} \end{aligned}$$

In the Eq. 1, $\textbf{q}^{\text {BN}}$ expresses the status of frame $\text {B}$ with respect to frame $\text {N}$. It is shown that the relationship of the vector of quaternions with $[\tilde{\varvec{\omega }}^{\text {BI}}]^{\text {B}}$ is as follows^39^:

2 $$\begin{aligned} \begin{bmatrix} \dot{q}_{0} \\ \dot{q}_{1} \\ \dot{q}_{2} \\ \dot{q}_{3} \\ \end{bmatrix}=0.5\, \begin{bmatrix} 0 &{} -p &{} -q &{} -r \\ p &{} 0 &{} r &{} -q \\ q &{} -r &{} 0 &{} p \\ r &{} q &{} -p &{} 0 \\ \end{bmatrix}\ \begin{bmatrix} {{q}_{0}} \\ {{q}_{1}} \\ {{q}_{2}} \\ {{q}_{3}} \\ \end{bmatrix} \end{aligned}$$

Where in the stationary condition we have:

3 $$\begin{aligned} \begin{array}{l} {{\varvec{\omega }}^{{\textrm{BN}}}} = {{\varvec{\omega }}^{{\textrm{BI}}}} - {{\varvec{\omega }}^{{\textrm{NE}}}} - {{\varvec{\omega }}^{{\textrm{EI}}}} \rightarrow \\ {[{{\varvec{\omega }}^{{\textrm{BN}}}}]^{\textrm{B}}} = {[{{\varvec{\omega }}^{{\textrm{BI}}}}]^{\textrm{B}}} - {[{\varvec{0}}]^{\textrm{B}}} - {[{{\varvec{\omega }}^{{\textrm{EI}}}}]^{\textrm{B}}} \rightarrow \\ \left[ {\begin{array}{*{20}{c}} p\\ q\\ r \end{array}} \right] = {[{{{\varvec{\tilde{\omega }}}}^{{\textrm{BI}}}}]^{\textrm{B}}} - [{\textbf{T}}]_{{\textrm{rough}}}^{{\textrm{BN}}}{[{{\varvec{\omega }}^{{\textrm{EI}}}}]^{\textrm{N}}} \end{array} \end{aligned}$$

In Eq. 3, $[\tilde{\varvec{\omega }}^{\text {BI}}]^{\text {B}}$ denotes gyroscope measurements and is modeled as follows:

4 $$\begin{aligned} \begin{bmatrix} \tilde{\omega }_\text {x} \\ \tilde{\omega }_\text {y} \\ \tilde{\omega }_\text {z} \end{bmatrix} = \begin{bmatrix} \text {s}_{x}^\text {g} &{} \text {m}_{\text {xy}}^\text {g} &{} \text {m}_{\text {xz}}^\text {g} \\ \text {m}_{\text {yx}}^\text {g} &{} \text {s}_{y}^\text {g} &{} \text {m}_{\text {yz}}^\text {g} \\ \text {m}_{\text {zx}}^\text {g} &{} \text {m}_{\text {zy}}^\text {g} &{} \text {s}_{z}^\text {g} \end{bmatrix} \begin{bmatrix} {\omega }_\text {x} \\ {\omega }_\text {y} \\ {\omega }_\text {z} \end{bmatrix} + \begin{bmatrix} \text {b}_{x}^\text {g}\\ \text {b}_{y}^\text {g} \\ \text {b}_{z}^\text {g} \end{bmatrix} + \begin{bmatrix} n_\text {x}^\text {g} \\ n_\text {y}^\text {g} \\ n_\text {z}^\text {g} \end{bmatrix} \end{aligned}$$

Based on the Eq. 4, the gyroscope error model includes misalignment coefficients that are denoted as ($\text {m}_{\text {xy}}^\text {g}$, $\text {m}_{\text {xz}}^\text {g}$, $\text {m}_{\text {yx}}^\text {g}$, $\text {m}_{\text {yz}}^\text {g}$, $\text {m}_{\text {zx}}^\text {g}$, $\text {m}_{\text {zy}}^\text {g}$), and scale factor coefficients for the $\text {x}$, $\text {y}$, and $\text {z}$ axes are denoted as ($\text {s}_{x}^\text {g}$, $\text {s}_{y}^\text {g}$, $\text {s}_{z}^\text {g}$) respectively. The bias error coefficients for the $\text {x}$, $\text {y}$, and $\text {z}$ axes are represented as ($\text {b}_{x}^\text {g}$, $\text {b}_{y}^\text {g}$, $\text {b}_{z}^\text {g}$). Additionally, the noise components for the $\text {x}$, $\text {y}$, and $\text {z}$ axes are denoted as ($n_\text {x}^\text {g}$, $n_\text {y}^\text {g}$, $n_\text {z}^\text {g}$).

In addition $[\varvec{\omega }^{\text {EI}}]^{\text {N}}$ is Earth rotation rate with respect to inertial frame and expressed in the navigation frame and $[\textbf{T}]^{\text {BN}}_{\textrm{rough}}$ is the initial guess of transformation matrix from navigation frame to body frame (provided by first phase course alignment block). Providing initial conditions for the quaternion vector, performed as follows:

5 $$\begin{aligned} \begin{aligned}{}&{{\left. {{q}_{0}} \right| }_{t=0}}=\cos ({{{\psi }_{0}}}/{2}\;)\cos ({{{\theta }_{0}}}/{2}\;)\cos ({{{\varphi }_{0}}}/{2}\;)+\sin ({{{\psi }_{0}}}/{2}\;)\sin ({{{\theta }_{0}}}/{2}\;)\sin ({{{\varphi }_{0}}}/{2}\;) \\&{{\left. {{q}_{1}} \right| }_{t=0}}=\cos ({{{\psi }_{0}}}/{2}\;)\cos ({{{\theta }_{0}}}/{2}\;)\sin ({{{\varphi }_{0}}}/{2}\;)-\sin ({{{\psi }_{0}}}/{2}\;)\sin ({{{\theta }_{0}}}/{2}\;)\cos ({{{\varphi }_{0}}}/{2}\;) \\&{{\left. {{q}_{2}} \right| }_{t=0}}=\cos ({{{\psi }_{0}}}/{2}\;)\sin ({{{\theta }_{0}}}/{2}\;)\cos ({{{\varphi }_{0}}}/{2}\;)+\sin ({{{\psi }_{0}}}/{2}\;)\cos ({{{\theta }_{0}}}/{2}\;)\sin ({{{\varphi }_{0}}}/{2}\;) \\&{{\left. {{q}_{3}} \right| }_{t=0}}=\sin ({{{\psi }_{0}}}/{2}\;)\cos ({{{\theta }_{0}}}/{2}\;)\cos ({{{\varphi }_{0}}}/{2}\;)-\cos ({{{\psi }_{0}}}/{2}\;)\sin ({{{\theta }_{0}}}/{2}\;)\sin ({{{\varphi }_{0}}}/{2}\;) \end{aligned} \end{aligned}$$

Where ${\varphi }_{0}$, ${\theta }_{0}$ and ${\psi }_{0}$ are provided by $[\text {T}]^{\text {BN}}_{\textrm{rough}}$ through the first phase coarse alignment block.

#### Attitude error calculator

The attitude error calculation block provides an estimation of attitude errors by using quaternion multiplication. The quaternion error vector is defined as follows:

6 $$\begin{aligned} {\hat{\textbf{q}}}=\textbf{q}\otimes {\tilde{\textbf{q}}}\rightarrow {\tilde{\textbf{q}}}={{\textbf{q}}^{-1}}\otimes {\hat{\textbf{q}}} \end{aligned}$$

Where $\textbf{q}$ is the true vector of quaternion, ${\hat{\textbf{q}}}$ is the propagated quaternion vector provided by quaternion propagation block in Fig. 2 and ${\tilde{\textbf{q}}}$ is the quaternion error vector which is the input of ‘Attitude Error Estimation’ block. In Eq. 6, symbol $\otimes$ is the quaternion multiplication operator as described in^40^ and ${{\textbf{q}}^{-1}}$ is provided by the first phase Course Alignment block and is defined as follows :

7 $$\begin{aligned} {{\textbf{q}}^{\text {-1}}}=\left[ \begin{matrix} {{q}_{0}} \\ -{{q}_{1}} \\ -{{q}_{2}} \\ -{{q}_{3}} \\ \end{matrix} \right] \end{aligned}$$

#### Quaternion-based bias calculator

The bias functions block in Fig. 2 calculates gyro biases through a series of analytical equations. The deviation in the attitude quaternion is defined as follows (Supplementary Information):

8 $$\begin{aligned} {{\tilde{\textbf{q}}}} = {{\textbf{q}}^{ - 1}} \otimes {{\hat{\textbf{q}}}} \end{aligned}$$

The time derivative of deviation quaternion is obtained by the chain rule as follows:

9 $$\begin{aligned} \dot{\tilde{\textbf{q}}} = \left( {\dot{\textbf{q}}}^{-1}\right) \otimes {\hat{\textbf{q}}} + \left( \textbf{q}^{-1}\right) \otimes {\dot{\hat{\textbf{q}}}} \end{aligned}$$

Also, the time derivative of the inverse quaternion is obtained as follows^41^:

10 $$\begin{aligned} \begin{array}{l} {\textbf{q}} \otimes {{\textbf{q}}^{ - 1}} = {\left[ {\begin{array}{*{20}{c}} 1&{}0&{}0&{}0 \end{array}} \right] ^{\textrm{T}}} \rightarrow {{\dot{\textbf{q}}}} \otimes {{\textbf{q}}^{ - 1}} + {\textbf{q}} \otimes {{{{\dot{\textbf{q}}}}}^{ - 1}} = {\textbf{0}} \rightarrow \\ {\textbf{q}} \otimes {{{{\dot{\textbf{q}}}}}^{ - 1}} = - {{\dot{\textbf{q}}}} \otimes {{\textbf{q}}^{ - 1}} \rightarrow {{{{\dot{\textbf{q}}}}}^{ - 1}} = - {{\textbf{q}}^{ - 1}} \otimes {{\dot{\textbf{q}}}} \otimes {{\textbf{q}}^{ - 1}} \end{array} \end{aligned}$$

Utilizing Eqs. 9 and 10, 9 is written:

11 $$\begin{aligned} \dot{\tilde{\textbf{q}}} = -\left( \textbf{q}^{-1} \otimes {\dot{\textbf{q}}} \otimes \textbf{q}^{-1} \otimes {\hat{\textbf{q}}}\right) + \left( \textbf{q}^{-1} \otimes {\dot{\hat{\textbf{q}}}}\right) \end{aligned}$$

The kinematic differential equation for the quaternion ${{\hat{\textbf{q}}}}$ is represented by:

12 $$\begin{aligned} {\begin{aligned} {\dot{\textbf{q}}}&= 0.5\left( \textbf{q} \otimes \begin{bmatrix} 0 \\ {[{\varvec{\omega }}^{\textrm{BN}}]^{\textrm{B}}}\end{bmatrix}\right) \\ \Rightarrow \varvec{\dot{\hat{q}}}&= 0.5 \left( {\hat{\textbf{q}}} \otimes \begin{bmatrix} 0 \\ {[{\varvec{\omega }}^{\textrm{BN}}]^{\textrm{B}} + [\delta {\varvec{\omega }}^{\textrm{BN}}]^{\textrm{B}}}\end{bmatrix}\right) \end{aligned}} \end{aligned}$$

Substituting Eq. 12 into Eq. 11 and utilizing ${{\hat{\textbf{q}}}} = {\textbf{q}} \otimes {{\tilde{\textbf{q}}}}$, $\dot{\tilde{\textbf{q}}}$ is obtained as follows^40^:

13 $$\begin{aligned} \begin{aligned} \dot{\tilde{\textbf{q}}}&= -0.5\left( \textbf{q}^{-1} \otimes \textbf{q} \otimes \begin{bmatrix} 0 \\ {[{\varvec{\omega }}^{\textrm{BN}}]^{\textrm{B}}}\end{bmatrix} \otimes \textbf{q}^{-1} \otimes \textbf{q} \otimes {\tilde{\textbf{q}}}\right) \\&\quad + 0.5\left( \textbf{q}^{-1} \otimes \textbf{q} \otimes {\tilde{\textbf{q}}} \otimes \begin{bmatrix} 0 \\ {[{\varvec{\omega }}^{\textrm{BN}}]^{\textrm{B}} + [\delta {\varvec{\omega }}^{\textrm{BN}}]^{\textrm{B}}}\end{bmatrix}\right) \\&= -0.5\begin{bmatrix} 0 \\ {[{\varvec{\omega }}^{\textrm{BN}}]^{\textrm{B}}}\end{bmatrix} \otimes {\tilde{\textbf{q}}} + 0.5 {\tilde{\textbf{q}}} \otimes \begin{bmatrix} 0 \\ {[{\varvec{\omega }}^{\textrm{BN}}]^{\textrm{B}} + [\delta {\varvec{\omega }}^{\textrm{BN}}]^{\textrm{B}}}\end{bmatrix} \\&= 0.5\begin{bmatrix} 0 \\ {[\delta {\varvec{\omega }}^{\textrm{BN}}]^{\textrm{B}}}\end{bmatrix} \otimes {\tilde{\textbf{q}}} \end{aligned} \end{aligned}$$

The above equation is rewritten as:

14 $$\begin{aligned} \dot{\tilde{\textbf{q}}} = 0.5\begin{bmatrix} 0 &{} -{[\delta {\bar{\varvec{\omega }}}^{\textrm{BI}}]^{\textrm{B}}} \\ {[\delta {\varvec{\omega }}^{\textrm{BI}}]^{\textrm{B}}} &{} {[\delta {\varvec{\Omega }}^{\textrm{BI}}]^{\textrm{B}}} \end{bmatrix} {\tilde{\textbf{q}}} \end{aligned}$$

Where ${{[\delta {{{{\bar{\varvec{\omega }}}}}^{\text {BN}}}]}^{\text {B}}}$ and ${{[\delta {{\mathbf{\Omega }}^{\text {BN}}}]}^{\text {B}}}$ are respectively transpose and skew-symmetric form of gyro error vector ${{[\delta {{{\varvec{\omega }}}^{\text {BN}}}]}^{\text {B}}}$ which is defined in the stationary condition as follows:

15 $$\begin{aligned} \begin{array}{l} {\mathrm{\delta }}{{\mathbf{\omega }}^{{\textrm{BN}}}} = {\mathrm{\delta }}{{\mathbf{\omega }}^{{\textrm{BI}}}} - {\mathrm{\delta }}{{\mathbf{\omega }}^{{\textrm{NE}}}} - {\mathrm{\delta }}{{\mathbf{\omega }}^{{\textrm{EI}}}} \rightarrow \\ {[{\mathrm{\delta }}{{\mathbf{\omega }}^{{\textrm{BN}}}}]^{\textrm{B}}} = {[{\mathrm{\delta }}{{\mathbf{\omega }}^{{\textrm{BI}}}}]^{\textrm{B}}} - {[{\mathrm{\delta }}{{\mathbf{\omega }}^{{\textrm{NE}}}}]^{\textrm{B}}} - {[{\mathrm{\delta }}{{\mathbf{\omega }}^{{\textrm{EI}}}}]^{\textrm{B}}} = {[{\mathrm{\delta }}{{\mathbf{\omega }}^{{\textrm{BI}}}}]^{\textrm{B}}} = \left[ {\begin{array}{*{20}{c}} {{\mathrm{\delta }}{\omega _{\textrm{x}}}}\\ {{\mathrm{\delta }}{\omega _{\textrm{y}}}}\\ {{\mathrm{\delta }}{\omega _{\textrm{z}}}} \end{array}} \right] \end{array} \end{aligned}$$

By substituting Eq. 15 into Eq. 14 (which is used for propagation of attitude error not the attitude), we will have:

16 $$\begin{aligned} \left[ \begin{matrix} {{{\dot{\tilde{q}}}}_{0}} \\ {{{\dot{\tilde{q}}}}_{1}} \\ {{{\dot{\tilde{q}}}}_{2}} \\ {{{\dot{\tilde{q}}}}_{3}} \\ \end{matrix} \right] =0.5\,\left[ \begin{matrix} 0 &{} -\text { }\!\!\delta \!\!\text { }{{\omega }_{\text {x}}} &{} -\text { }\!\!\delta \!\!\text { }{{\omega }_{\text {y}}} &{} -\text { }\!\!\delta \!\!\text { }{{\omega }_{\text {z}}} \\ \text { }\!\!\delta \!\!\text { }{{\omega }_{\text {x}}} &{} 0 &{} -\text { }\!\!\delta \!\!\text { }{{\omega }_{\text {z}}} &{} \text { }\!\!\delta \!\!\text { }{{\omega }_{\text {y}}} \\ \text { }\!\!\delta \!\!\text { }{{\omega }_{\text {y}}} &{} \text { }\!\!\delta \!\!\text { }{{\omega }_{\text {z}}} &{} 0 &{} -\text { }\!\!\delta \!\!\text { }{{\omega }_{\text {x}}} \\ \text { }\!\!\delta \!\!\text { }{{\omega }_{\text {z}}} &{} -\text { }\!\!\delta \!\!\text { }{{\omega }_{\text {y}}} &{} \text { }\!\!\delta \!\!\text { }{{\omega }_{\text {x}}} &{} 0 \\ \end{matrix} \right] \,\left[ \begin{matrix} {{{\tilde{q}}}_{0}} \\ {{{\tilde{q}}}_{1}} \\ {{{\tilde{q}}}_{2}} \\ {{{\tilde{q}}}_{3}} \\ \end{matrix} \right] \end{aligned}$$

The expansion of the above equation is as follows:

17 $$\begin{aligned} \begin{aligned}{}&{{{\dot{\tilde{q}}}}_{0}}=0.5(-\text { }\!\!\delta \!\!\text { }{{\omega }_{\text {x}}}{{{\tilde{q}}}_{1}}-\text { }\!\!\delta \!\!\text { }{{\omega }_{\text {y}}}{{{\tilde{q}}}_{2}}-\text { }\!\!\delta \!\!\text { }{{\omega }_{\text {z}}}{{{\tilde{q}}}_{3}}) \\&{{{\dot{\tilde{q}}}}_{1}}=0.5(+\text { }\!\!\delta \!\!\text { }{{\omega }_{\text {x}}}{{{\tilde{q}}}_{0}}-\text { }\!\!\delta \!\!\text { }{{\omega }_{\text {z}}}{{{\tilde{q}}}_{2}}+\text { }\!\!\delta \!\!\text { }{{\omega }_{\text {y}}}{{{\tilde{q}}}_{3}}) \\&{{{\dot{\tilde{q}}}}_{2}}=0.5(+\text { }\!\!\delta \!\!\text { }{{\omega }_{\text {y}}}{{{\tilde{q}}}_{0}}+\text { }\!\!\delta \!\!\text { }{{\omega }_{\text {z}}}{{{\tilde{q}}}_{1}}-\text { }\!\!\delta \!\!\text { }{{\omega }_{\text {x}}}{{{\tilde{q}}}_{3}}) \\&{{{\dot{\tilde{q}}}}_{3}}=0.5(+\text { }\!\!\delta \!\!\text { }{{\omega }_{\text {z}}}{{{\tilde{q}}}_{0}}-\text { }\!\!\delta \!\!\text { }{{\omega }_{\text {y}}}{{{\tilde{q}}}_{1}}+\text { }\!\!\delta \!\!\text { }{{\omega }_{\text {x}}}{{{\tilde{q}}}_{2}}) \\ \end{aligned} \end{aligned}$$

The initial conditions of the above differential equations are obtained from the first phase coarse alignment introduced in Fig. 2. Integrating the above relations, results in:

18 $$\begin{aligned} \begin{aligned}{}&\int _{0}^{{{\text {t}}_{\text {f}}}}{{{{\dot{\tilde{q}}}}_{0}}\text {(}t\text {)d}t}=-0.5\text { }\!\!\delta \!\!\text { }{{\omega }_{\text {x}}}\int _{0}^{{{\text {t}}_{\text {f}}}}{{{{\tilde{q}}}_{1}}\text {(}t\text {)d}t}-0.5\text { }\!\!\delta \!\!\text { }{{\omega }_{\text {y}}}\int _{0}^{{{\text {t}}_{\text {f}}}}{{{{\tilde{q}}}_{2}}\text {(}t\text {)d}t}-0.5\text 
{ }\!\!\delta \!\!\text { }{{\omega }_{\text {z}}}\int _{0}^{{{\text {t}}_{\text {f}}}}{{{{\tilde{q}}}_{3}}\text {(}t\text {)d}t} \\&\int _{0}^{{{\text {t}}_{\text {f}}}}{{{{\dot{\tilde{q}}}}_{1}}\text {(}t\text {)d}t}=0.5\text { }\!\!\delta \!\!\text { }{{\omega }_{\text {x}}}\int _{0}^{{{\text {t}}_{\text {f}}}}{{{{\tilde{q}}}_{0}}\text {(}t\text {)d}t}-0.5\text { }\!\!\delta \!\!\text { }{{\omega }_{\text {z}}}\int _{0}^{{{\text {t}}_{\text {f}}}}{{{{\tilde{q}}}_{2}}\text {(}t\text {)d}t}+0.5\text { }\!\!\delta \!\!\text { }{{\omega }_{\text {y}}}\int _{0}^{{{\text {t}}_{\text {f}}}}{{{{\tilde{q}}}_{3}}\text {(}t\text {)d}t} \\&\int _{0}^{{{\text {t}}_{\text {f}}}}{{{{\dot{\tilde{q}}}}_{2}}\text {(}t\text {)d}t}=0.5\text { }\!\!\delta \!\!\text { }{{\omega }_{\text {y}}}\int _{0}^{{{\text {t}}_{\text {f}}}}{{{{\tilde{q}}}_{0}}\text {(}t\text {)d}t}+0.5\text { }\!\!\delta \!\!\text { }{{\omega }_{\text {z}}}\int _{0}^{{{\text {t}}_{\text {f}}}}{{{{\tilde{q}}}_{1}}\text {(}t\text {)d}t}-0.5\text { }\!\!\delta \!\!\text { }{{\omega }_{\text {x}}}\int _{0}^{{{\text {t}}_{\text {f}}}}{{{{\tilde{q}}}_{3}}\text {(}t\text {)d}t} \\&\int _{0}^{{{\text {t}}_{\text {f}}}}{{{{\dot{\tilde{q}}}}_{3}}\text {(}t\text {)d}t}=0.5\text { }\!\!\delta \!\!\text { }{{\omega }_{\text {z}}}\int _{0}^{{{\text {t}}_{\text {f}}}}{{{{\tilde{q}}}_{0}}\text {(}t\text {)d}t}-0.5\text { }\!\!\delta \!\!\text { }{{\omega }_{\text {y}}}\int _{0}^{{{\text {t}}_{\text {f}}}}{{{{\tilde{q}}}_{1}}\text {(}t\text {)d}t}+0.5\text { }\!\!\delta \!\!\text { }{{\omega }_{\text {x}}}\int _{0}^{{{\text {t}}_{\text {f}}}}{{{{\tilde{q}}}_{2}}\text {(}t\text {)d}t} \\ \end{aligned} \end{aligned}$$

The above relation is rewritten as follows:

19 $$\begin{aligned} \begin{aligned}{}&-\text { }\!\!\delta \!\!\text { }{{\omega }_{\text {x}}}\int _{0}^{{{\text {t}}_{\text {f}}}}{{{{\tilde{q}}}_{1}}\text {(}t\text {)d}t}-\text { }\!\!\delta \!\!\text { }{{\omega }_{\text {y}}}\int _{0}^{{{\text {t}}_{\text {f}}}}{{{{\tilde{q}}}_{2}}\text {(}t\text {)d}t}-\text { }\!\!\delta \!\!\text { }{{\omega }_{\text {z}}}\int _{0}^{{{\text {t}}_{\text {f}}}}{{{{\tilde{q}}}_{3}}\text {(}t\text {)d}t}=2{{{\tilde{q}}}_{0}}\text {(}{{\text {t}}_{\text {f}}}\text {)}-2{{{\tilde{q}}}_{0}}\text {(0)} \\&\text { }\!\!\delta \!\!\text { }{{\omega }_{\text {x}}}\int _{0}^{{{\text {t}}_{\text {f}}}}{{{{\tilde{q}}}_{0}}\text {(}t\text {)d}t}+\text { }\!\!\delta \!\!\text { }{{\omega }_{\text {y}}}\int _{0}^{{{\text {t}}_{\text {f}}}}{{{{\tilde{q}}}_{3}}\text {(}t\text {)d}t}-\text { }\!\!\delta \!\!\text { }{{\omega }_{\text {z}}}\int _{0}^{{{\text {t}}_{\text {f}}}}{{{{\tilde{q}}}_{2}}\text {(}t\text {)d}t}=2{{{\tilde{q}}}_{1}}\text {(}{{\text {t}}_{\text {f}}}\text {)}-2{{{\tilde{q}}}_{1}}\text {(0)} \\&-\text { }\!\!\delta \!\!\text { }{{\omega }_{\text {x}}}\int _{0}^{{{\text {t}}_{\text {f}}}}{{{{\tilde{q}}}_{3}}\text {(}t\text {)d}t}+\text { }\!\!\delta \!\!\text { }{{\omega }_{\text {y}}}\int _{0}^{{{\text {t}}_{\text {f}}}}{{{{\tilde{q}}}_{0}}\text {(}t\text {)d}t}+\text { }\!\!\delta \!\!\text { }{{\omega }_{\text {z}}}\int _{0}^{{{\text {t}}_{\text {f}}}}{{{{\tilde{q}}}_{1}}\text {(}t\text {)d}t}=2{{{\tilde{q}}}_{2}}\text {(}{{\text {t}}_{\text {f}}}\text {)}-2{{{\tilde{q}}}_{2}}\text {(0)} \\&\text { }\!\!\delta \!\!\text { }{{\omega }_{\text {x}}}\int _{0}^{{{\text {t}}_{\text {f}}}}{{{{\tilde{q}}}_{2}}\text {(}t\text {)d}t}-\text { }\!\!\delta \!\!\text { }{{\omega }_{\text {y}}}\int _{0}^{{{\text {t}}_{\text {f}}}}{{{{\tilde{q}}}_{1}}\text {(}t\text {)d}t}+\text { }\!\!\delta \!\!\text { }{{\omega }_{\text {z}}}\int _{0}^{{{\text {t}}_{\text {f}}}}{{{{\tilde{q}}}_{0}}\text {(}t\text {)d}t}=2{{{\tilde{q}}}_{3}}\text {(}{{\text {t}}_{\text {f}}}\text {)}-2{{{\tilde{q}}}_{3}}\text {(0)} \\ \end{aligned} \end{aligned}$$

The above relation includes four equations and three unknowns and is rewritten as:

20 $$\begin{aligned} {\textbf{U}\theta }=\textbf{y} \end{aligned}$$

Where

21 $$\begin{aligned} \textbf{U}=\left[ \begin{matrix} -\int _{0}^{{{\text {t}}_{\text {f}}}}{{{{\tilde{q}}}_{1}}\text {(}t\text {)d}t} &{} -\int _{0}^{{{\text {t}}_{\text {f}}}}{{{{\tilde{q}}}_{2}}\text {(}t\text {)d}t} &{} -\int _{0}^{{{\text {t}}_{\text {f}}}}{{{{\tilde{q}}}_{3}}\text {(}t\text {)d}t} \\ +\int _{0}^{{{\text {t}}_{\text {f}}}}{{{{\tilde{q}}}_{0}}\text {(}t\text {)d}t} &{} +\int _{0}^{{{\text {t}}_{\text {f}}}}{{{{\tilde{q}}}_{3}}\text {(}t\text {)d}t} &{} -\int _{0}^{{{\text {t}}_{\text {f}}}}{{{{\tilde{q}}}_{2}}\text {(}t\text {)d}t} \\ -\int _{0}^{{{\text {t}}_{\text {f}}}}{{{{\tilde{q}}}_{3}}\text {(}t\text {)d}t} &{} +\int _{0}^{{{\text {t}}_{\text {f}}}}{{{{\tilde{q}}}_{0}}\text {(}t\text {)d}t} &{} +\int _{0}^{{{\text {t}}_{\text {f}}}}{{{{\tilde{q}}}_{1}}\text {(}t\text {)d}t} \\ +\int _{0}^{{{\text {t}}_{\text {f}}}}{{{{\tilde{q}}}_{2}}\text {(}t\text {)d}t} &{} -\int _{0}^{{{\text {t}}_{\text {f}}}}{{{{\tilde{q}}}_{1}}\text {(}t\text {)d}t} &{} +\int _{0}^{{{\text {t}}_{\text {f}}}}{{{{\tilde{q}}}_{0}}\text {(}t\text {)d}t} \\ \end{matrix} \right] \end{aligned}$$

and

22 $$\begin{aligned} {\varvec{\theta }}=\left[ \begin{array}{l} \text { }\!\!\delta \!\!\text { }{{\omega }_{\text {x}}} \\ \text { }\!\!\delta \!\!\text { }{{\omega }_{\text {y}}} \\ \text { }\!\!\delta \!\!\text { }{{\omega }_{\text {z}}} \\ \end{array} \right] \end{aligned}$$

and

23 $$\begin{aligned} \textbf{y}=2\left[ \begin{matrix} {{{\tilde{q}}}_{0}}\text {(}{{\text {t}}_{\text {f}}}\text {)}-{{{\tilde{q}}}_{0}}\text {(0)} \\ {{{\tilde{q}}}_{1}}\text {(}{{\text {t}}_{\text {f}}}\text {)}-{{{\tilde{q}}}_{1}}\text {(0)} \\ {{{\tilde{q}}}_{2}}\text {(}{{\text {t}}_{\text {f}}}\text {)}-{{{\tilde{q}}}_{2}}\text {(0)} \\ {{{\tilde{q}}}_{3}}\text {(}{{\text {t}}_{\text {f}}}\text {)}-{{{\tilde{q}}}_{3}}\text {(0)} \\ \end{matrix} \right] =2\left[ \begin{matrix} {{{\tilde{q}}}_{0}}\text {(}{{\text {t}}_{\text {f}}}\text {)}-1 \\ {{{\tilde{q}}}_{1}}\text {(}{{\text {t}}_{\text {f}}}\text {)} \\ {{{\tilde{q}}}_{2}}\text {(}{{\text {t}}_{\text {f}}}\text {)} \\ {{{\tilde{q}}}_{3}}\text {(}{{\text {t}}_{\text {f}}}\text {)} \\ \end{matrix} \right] \end{aligned}$$

Based on the least squares method, the solution of Eq. 20 is as follows^42^:

24 $$\begin{aligned} {\hat{\varvec{\theta }}}={{({{\textbf{U}}^{\text {T}}}\textbf{U})}^{-1}}{{\textbf{U}}^{\text {T}}}\textbf{y} \end{aligned}$$

By substituting Eqs. 21, 22 and 23 in Eq. 20 and solving the resulted equation through Eq. 24, we would have:

25 $$\begin{aligned}{} & {} \text { }\!\!\delta \!\!\text { }{\hat{{\omega }}_{\text {x}}}=\text {2}\frac{\left( \text {1}-{{{\tilde{q}}}_{0}}\text {(}{{\text {t}}_{\text {f}}}\text {)} \right) \int _{0}^{{{\text {t}}_{\text {f}}}}{{{{\tilde{q}}}_{1}}\text {(}t\text {)d}t}+{{{\tilde{q}}}_{1}}\text {(}{{\text {t}}_{\text {f}}}\text {)}\int _{0}^{{{\text {t}}_{\text {f}}}}{{{{\tilde{q}}}_{0}}\text {(}t\text {)d}t}-{{{\tilde{q}}}_{2}}\text {(}{{\text {t}}_{\text {f}}}\text {)}\int _{0}^{{{\text {t}}_{\text {f}}}}{{{{\tilde{q}}}_{3}}\text {(}t\text {)d}t}+{{{\tilde{q}}}_{3}}\text {(}{{\text {t}}_{\text {f}}}\text {)}\int _{0}^{{{\text {t}}_{\text {f}}}}{{{{\tilde{q}}}_{2}}\text {(}t\text {)d}t}}{{{\left( \int _{0}^{{{\text {t}}_{\text {f}}}}{{{{\tilde{q}}}_{0}}\text {(}t\text {)d}t} \right) }^{\text {2}}}+{{\left( \int _{0}^{{{\text {t}}_{\text {f}}}}{{{{\tilde{q}}}_{1}}\text {(}t\text {)d}t} \right) }^{\text {2}}}+{{\left( \int _{0}^{{{\text {t}}_{\text {f}}}}{{{{\tilde{q}}}_{2}}\text {(}t\text {)d}t} \right) }^{\text {2}}}+{{\left( \int _{0}^{{{\text {t}}_{\text {f}}}}{{{{\tilde{q}}}_{3}}\text {(}t\text {)d}t} \right) }^{\text {2}}}} \end{aligned}$$

26 $$\begin{aligned}{} & {} \text { }\!\!\delta \!\!\text { }{\hat{{\omega }}_{\text {y}}}=\text {2}\frac{\left( \text {1}-{{{\tilde{q}}}_{0}}\text {(}{{\text {t}}_{\text {f}}}\text {)} \right) \int _{0}^{{{\text {t}}_{\text {f}}}}{{{{\tilde{q}}}_{2}}\text {(}t\text {)d}t}+{{{\tilde{q}}}_{1}}\text {(}{{\text {t}}_{\text {f}}}\text {)}\int _{0}^{{{\text {t}}_{\text {f}}}}{{{{\tilde{q}}}_{3}}\text {(}t\text {)d}t}+{{{\tilde{q}}}_{2}}\text {(}{{\text {t}}_{\text {f}}}\text {)}\int _{0}^{{{\text {t}}_{\text {f}}}}{{{{\tilde{q}}}_{0}}\text {(}t\text {)d}t}-{{{\tilde{q}}}_{3}}\text {(}{{\text {t}}_{\text {f}}}\text {)}\int _{0}^{{{\text {t}}_{\text {f}}}}{{{{\tilde{q}}}_{1}}\text {(}t\text {)d}t}}{{{\left( \int _{0}^{{{\text {t}}_{\text {f}}}}{{{{\tilde{q}}}_{0}}\text {(}t\text {)d}t} \right) }^{\text {2}}}+{{\left( \int _{0}^{{{\text {t}}_{\text {f}}}}{{{{\tilde{q}}}_{1}}\text {(}t\text {)d}t} \right) }^{\text {2}}}+{{\left( \int _{0}^{{{\text {t}}_{\text {f}}}}{{{{\tilde{q}}}_{2}}\text {(}t\text {)d}t} \right) }^{\text {2}}}+{{\left( \int _{0}^{{{\text {t}}_{\text {f}}}}{{{{\tilde{q}}}_{3}}\text {(}t\text {)d}t} \right) }^{\text {2}}}} \end{aligned}$$

27 $$\begin{aligned}{} & {} \text { }\!\!\delta \!\!\text { }{\hat{{\omega }}_{\text {z}}}=\text {2}\frac{\left( \text {1}-{{{\tilde{q}}}_{0}}\text {(}{{\text {t}}_{\text {f}}}\text {)} \right) \int _{0}^{{{\text {t}}_{\text {f}}}}{{{{\tilde{q}}}_{3}}\text {(}t\text {)d}t}-{{{\tilde{q}}}_{1}}\text {(}{{\text {t}}_{\text {f}}}\text {)}\int _{0}^{{{\text {t}}_{\text {f}}}}{{{{\tilde{q}}}_{2}}\text {(}t\text {)d}t}+{{{\tilde{q}}}_{2}}\text {(}{{\text {t}}_{\text {f}}}\text {)}\int _{0}^{{{\text {t}}_{\text {f}}}}{{{{\tilde{q}}}_{1}}\text {(}t\text {)d}t}+{{{\tilde{q}}}_{3}}\text {(}{{\text {t}}_{\text {f}}}\text {)}\int _{0}^{{{\text {t}}_{\text {f}}}}{{{{\tilde{q}}}_{0}}\text {(}t\text {)d}t}}{{{\left( \int _{0}^{{{\text {t}}_{\text {f}}}}{{{{\tilde{q}}}_{0}}\text {(}t\text {)d}t} \right) }^{\text {2}}}+{{\left( \int _{0}^{{{\text {t}}_{\text {f}}}}{{{{\tilde{q}}}_{1}}\text {(}t\text {)d}t} \right) }^{\text {2}}}+{{\left( \int _{0}^{{{\text {t}}_{\text {f}}}}{{{{\tilde{q}}}_{2}}\text {(}t\text {)d}t} \right) }^{\text {2}}}+{{\left( \int _{0}^{{{\text {t}}_{\text {f}}}}{{{{\tilde{q}}}_{3}}\text {(}t\text {)d}t} \right) }^{\text {2}}}} \end{aligned}$$

Where $\delta {{\hat{\omega }}_{\textrm{x}}}$, $\delta {{\hat{\omega }}_{\textrm{y}}}$ and $\delta {{\hat{\omega }}_{\textrm{z}}}$ are estimated gyro biases of $\text {x}$, $\text {y}$ and $\text {z}$ axes respectively.

If ${\hat{\textbf{q}}}$ is assumed an acceptable approximation of $\textbf{q}$, the quaternion error vector can be written as follows:

28 $$\begin{aligned} {\tilde{\textbf{q}}}={{\textbf{q}}^{-1}}\otimes {\hat{\textbf{q}}}\approx {{\textbf{q}}^{-1}}\otimes \textbf{q}={{[\begin{matrix} 1 &{} 0 &{} 0 &{} 0 \\ \end{matrix}]}^{\text {T}}} \end{aligned}$$

Considering the assumption of Eq. 28, the denominator of the Eqs. 25, 26 and 27 will be simplified as follows:

29 $$\begin{aligned} \begin{aligned}{}&{{\left( \int _{0}^{{{\text {t}}_{\text {f}}}}{{{{\tilde{q}}}_{0}}\text {(}t\text {)d}t} \right) }^{\text {2}}}+{{\left( \int _{0}^{{{\text {t}}_{\text {f}}}}{{{{\tilde{q}}}_{1}}\text {(}t\text {)d}t} \right) }^{\text {2}}}+{{\left( \int _{0}^{{{\text {t}}_{\text {f}}}}{{{{\tilde{q}}}_{2}}\text {(}t\text {)d}t} \right) }^{\text {2}}}+{{\left( \int _{0}^{{{\text {t}}_{\text {f}}}}{{{{\tilde{q}}}_{3}}\text {(}t\text {)d}t} \right) }^{\text {2}}}\approx \\&{{\left( \int _{0}^{{{\text {t}}_{\text {f}}}}{1\text {d}t} \right) }^{\text {2}}}+{{\left( \int _{0}^{{{\text {t}}_{\text {f}}}}{0\text {d}t} \right) }^{\text {2}}}+{{\left( \int _{0}^{{{\text {t}}_{\text {f}}}}{0\text {d}t} \right) }^{\text {2}}}+{{\left( \int _{0}^{{{\text {t}}_{\text {f}}}}{0\text {d}t} \right) }^{\text {2}}}={{\left( \,\,[t]_{0}^{{{\text {t}}_{\text {f}}}} \right) }^{\text {2}}}={{\text {t}}_{\text {f}}}^{2} \\ \end{aligned} \end{aligned}$$

Where $\text {t}_\text {f}$ is the maximum time of integration that can be specified by user.

Finally by placing relation 29 in Eqs. 25–27, bias functions equations will be:

30 $$\begin{aligned}{} & {} { {\mathrm{\delta }}{{\hat{\omega }}_{\textrm{x}}} = {\textrm{2}}\frac{{\left( {{\textrm{1}} - {{\tilde{q}}_0}{\mathrm{(}}{{\textrm{t}}_{\textrm{f}}}{\mathrm{)}}} \right) \int _0^{{{\textrm{t}}_{\textrm{f}}}} {{{\tilde{q}}_1}{\mathrm{(}}t{\mathrm{)d}}t} + {{\tilde{q}}_1}{\mathrm{(}}{{\textrm{t}}_{\textrm{f}}}{\mathrm{)}}\int _0^{{{\textrm{t}}_{\textrm{f}}}} {{{{\tilde{q}}}_0}{\mathrm{(}}t{\mathrm{)d}}t} - {{\tilde{q}}_2}{\mathrm{(}}{{\textrm{t}}_{\textrm{f}}}{\mathrm{)}}\int _0^{{{\textrm{t}}_{\textrm{f}}}} {{{{\tilde{q}}}_3}{\mathrm{(}}t{\mathrm{)d}}t} + {{\tilde{q}}_3}{\mathrm{(}}{{\textrm{t}}_{\textrm{f}}}{\mathrm{)}}\int _0^{{{\textrm{t}}_{\textrm{f}}}} {{{{\tilde{q}}}_2}{\mathrm{(}}t{\mathrm{)d}}t} }}{{{{\textrm{t}}_{\textrm{f}}}^2}}} \end{aligned}$$

31 $$\begin{aligned}{} & {} { {\mathrm{\delta }}{{\hat{\omega }}_{\textrm{y}}} = {\textrm{2}}\frac{{\left( {{\textrm{1}} - {{\tilde{q}}_0}{\mathrm{(}}{{\textrm{t}}_{\textrm{f}}}{\mathrm{)}}} \right) \int _0^{{{\textrm{t}}_{\textrm{f}}}} {{{\tilde{q}}_2}{\mathrm{(}}t{\mathrm{)d}}t} + {{\tilde{q}}_1}{\mathrm{(}}{{\textrm{t}}_{\textrm{f}}}{\mathrm{)}}\int _0^{{{\textrm{t}}_{\textrm{f}}}} {{{{\tilde{q}}}_3}{\mathrm{(}}t{\mathrm{)d}}t} + {{\tilde{q}}_2}{\mathrm{(}}{{\textrm{t}}_{\textrm{f}}}{\mathrm{)}}\int _0^{{{\textrm{t}}_{\textrm{f}}}} {{{{\tilde{q}}}_0}{\mathrm{(}}t{\mathrm{)d}}t} - {{\tilde{q}}_3}{\mathrm{(}}{{\textrm{t}}_{\textrm{f}}}{\mathrm{)}}\int _0^{{{\textrm{t}}_{\textrm{f}}}} {{{{\tilde{q}}}_1}{\mathrm{(}}t{\mathrm{)d}}t} }}{{{{\textrm{t}}_{\textrm{f}}}^2}}} \end{aligned}$$

32 $$\begin{aligned}{} & {} { {\mathrm{\delta }}{{\hat{\omega }}_{\textrm{z}}} = {\textrm{2}}\frac{{\left( {{\textrm{1}} - {{\tilde{q}}_0}{\mathrm{(}}{{\textrm{t}}_{\textrm{f}}}{\mathrm{)}}} \right) \int _0^{{{\textrm{t}}_{\textrm{f}}}} {{{\tilde{q}}_3}{\mathrm{(}}t{\mathrm{)d}}t} - {{\tilde{q}}_1}{\mathrm{(}}{{\textrm{t}}_{\textrm{f}}}{\mathrm{)}}\int _0^{{{\textrm{t}}_{\textrm{f}}}} {{{{\tilde{q}}}_2}{\mathrm{(}}t{\mathrm{)d}}t} + {{\tilde{q}}_2}{\mathrm{(}}{{\textrm{t}}_{\textrm{f}}}{\mathrm{)}}\int _0^{{{\textrm{t}}_{\textrm{f}}}} {{{{\tilde{q}}}_1}{\mathrm{(}}t{\mathrm{)d}}t} + {{\tilde{q}}_3}{\mathrm{(}}{{\textrm{t}}_{\textrm{f}}}{\mathrm{)}}\int _0^{{{\textrm{t}}_{\textrm{f}}}} {{{{\tilde{q}}}_0}{\mathrm{(}}t{\mathrm{)d}}t} }}{{{{\textrm{t}}_{\textrm{f}}}^2}}} \end{aligned}$$

Equations 30–32 are embedded in the “Bias Functions” block as depicted in Fig. 2.

## Numerical simulation and experimental results

The extracted relationships are validated in this section through numerical simulation and experimental results. Firstly, the relationships are tested using numerical simulations to ensure their accuracy and reliability. Secondly, experiments are conducted to validate the extracted relationships further in a real-world.

### Numerical simulation

A comprehensive analysis was performed through numerical simulations to validate the accuracy and reliability of the proposed method; a total of 200 working points were generated for this purpose. These working points encompass randomly generated stationary conditions for the inertial block (latitude, longitude, height, Euler angles), and distinct error coefficients were applied to the gyro model based on the Eq. 4 at each scenario. Tables 1 and 2 demonstrate the first five working points of the simulation.

**Table 1:** Generated test conditions—Position and Attitude of the IMU.

Scenario number	Position			Attitude		
	$\lambda$ ($^{\circ }$)	$\ell$ ($^{\circ }$)	$h (\textrm{m})$	$\varphi$ ($^{\circ }$)	$\theta$ ($^{\circ }$)	$\psi$ ($^{\circ }$)
1	59.281	325.887	4934.841	56.129	79.545	− 21.372
2	− 60.778	98.827	874.46	− 24.985	81.842	− 170.601
3	78.465	271.845	36.743	147.275	73.835	39.109
4	4.991	295.494	4293.795	50.139	74.097	126.129
5	− 41.587	36.033	4191.278	61.727	− 65.914	− 65.482

**Table 2:** Generated test conditions–Gyro model coefficients.

Sn	Bias (°/hr)			Scale Factor (ppm)			Missalignment (arc second)					
	$b_{x}^{g}$	$b_{y}^{g}$	$b_{z}^{g}$	$s_{x}^{g}$	$s_{y}^{g}$	$s_{z}^{g}$	$m_{\text {xy}}^{g}$	$m_{\text {xz}}^{g}$	$m_{\text {yx}}^{g}$	$m_{\text {yz}}^{g}$	$m_{\text {zx}}^{g}$	$m_{\text {zy}}^{g}$
1	0.01	0.008	0.006	20	22	25	3	6	5	3	7	5
2	0.006	0.011	0.014	29	14	20	4	5	4	3	5	3
3	0.012	0.012	0.011	21	14	25	4	4	7	7	5	4
4	0.013	0.008	0.01	25	12	12	4	5	7	6	4	4
5	0.011	0.014	0.006	22	16	27	3	5	4	7	6	4

Next, the simulation is conducted using the set above of working points in stationary conditions. Firstly, the sensors’ output was averaged for 20 s while they were entirely at rest. Then, the proposed algorithm is executed for a duration of 1 second. the sampling rate of the quaternion propagation procedure is 100 Hz.

#### Accuracy analysis

**Figure 3:**
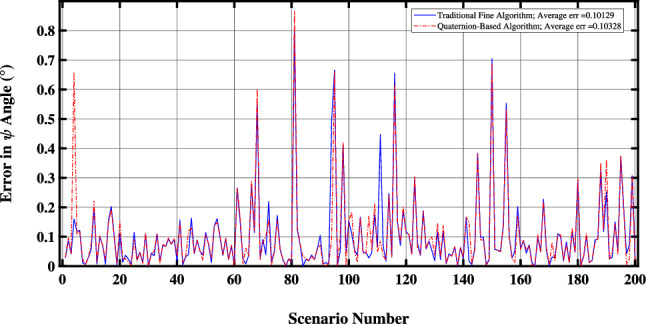
Accuracy comparison of fine and the proposed QBDA algorithms.

In Fig. 3, the accuracy of the proposed QBDA method is compared with the maximum achievable accuracy of traditional fine alignment methods in calculating the north direction. As depicted in Fig. 3, the accuracy of the QBDA method is comparable to that of conventional fine alignment algorithms.

**Figure 4:**
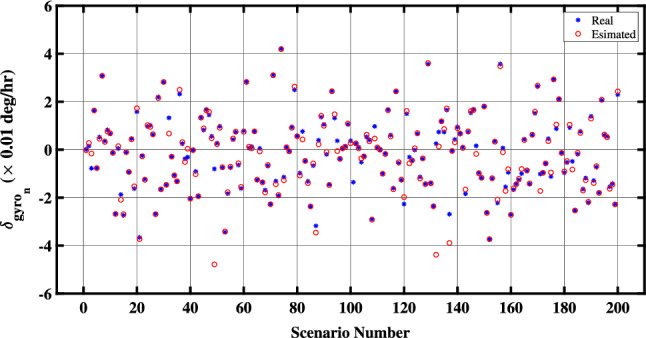
Estimation of the north-channel-gyro bias in the proposed QBDA algorithm.

**Figure 5:**
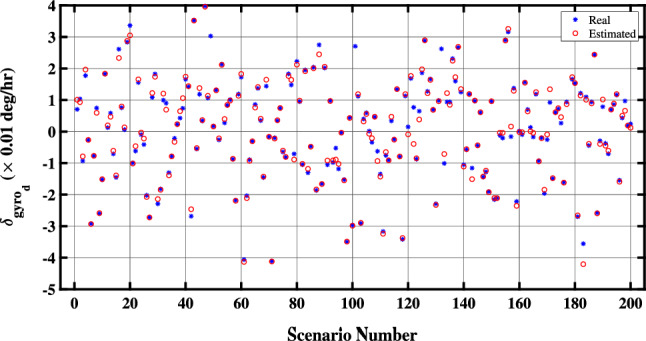
Estimation of the down-channel-gyro bias in the proposed QBDA algorithm.

In^15^, the observable and unobservable parameters in the fine alignment algorithm are specified. According to this reference, the unobservable parameters encompass the bias of north and east accelerometers, as well as the east gyroscope bias. Conversely, the north and down gyroscope biases are identified as observable. As a result, it is anticipated that the proposed method will demonstrate the capability to estimate both the north gyro bias ${\delta _{gn}}$ and the down gyro bias ${\delta _{gd}}$ as accurate as a conventional fine alignment algorithm. Figures 4 and 5 depict the effective estimation of the north gyro bias ${\delta _{gn}}$ and the down gyro bias ${\delta _{gd}}$ achieved through the QBDA method, respectively.

**Figure 6:**
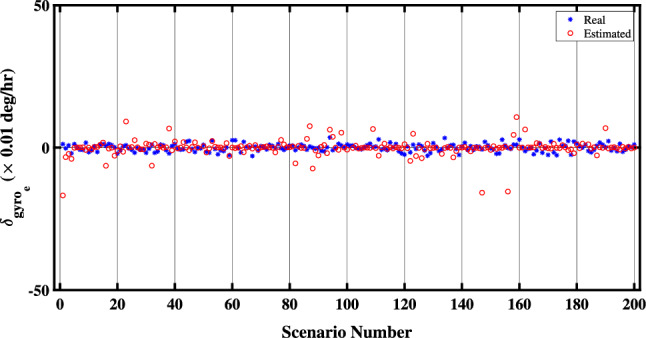
Estimation of the east-channel-gyro bias in the proposed QBDA algorithm.

Lastly, Figure 6 illustrates the QBDA method’s estimation of the east-channel-gyro bias (${\delta _{ge}}$). As previously indicated, ${\delta _{ge}}$ is unobservable; consequently, its estimation remains unattainable. Therefore, the differential alignment algorithm has approximated the value of this unobservable parameter as zero.

#### Execution time

The proposed algorithm comprises three distinct phases, with only phase 1 being time-intensive. During phase 1, the outputs of the IMU undergo an averaging process lasting approximately 20-30 s to mitigate the impact of environmental noise. This results in three averaged values for accelerometers and three for gyroscopes. Phases 2 and 3 are not time-intensive because they work with the average numbers prepared in Phase 1. The computational processes in phases 2 and 3 are performed in a fraction of time. The algorithm was run on a computing system equipped with an Intel Core i5 CPU running at 1.6GHz, coupled with 8GB of RAM, Table 3 presents the subsequent run-time procedure:

**Table 3:** run-time procedure.

Phase	Execution time (sec)
1	20
2	0.015
3	0.01
Sum	20.025

**Figure 7:**
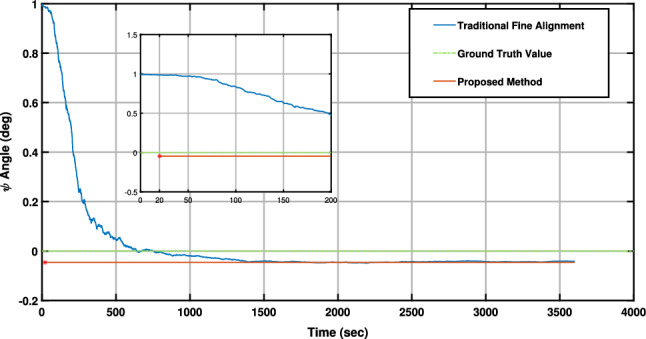
Convergence time comparison between the proposed method and a traditional Kalman filter-based fine alignment.

Figure 7 illustrates a comparison of convergence times between the proposed method and a traditional Kalman filter-based fine alignment. Notably, the QBDA method achieves an estimation of the north direction within 20 s, superior than the traditional fine alignment, which demands approximately 10 min to converge to the desired accuracy.

### Experimental results

The proposed alignment method was validated in real-world conditions using FOG100 GEM Elettronica gyrocompass data. This system includes a triad of fiber optical gyroscopes along with a triad of quartz accelerometers. Tables 4 and 5 represent gyroscopes and accelerometers specifications respectively.

**Table 4:** Gyroscope Specification.

Parameter	Unit	Value
Measurement range	deg/sec	±490
Bandwidth	Hz	400
Initial bias	deg/hr	$<0.02$
Bias instability	deg/hr	0.01
Initial scaling error	ppm	100
Scale factor stability	ppm	20
Non-linearity	ppm	10
Misalignment error	deg	$<0.001$
Noise density	${\mathrm{deg/hr/}}\sqrt{{\textrm{Hz}}}$	0.3
Angular random walk	${\mathrm{deg/}}\sqrt{{\textrm{hr}}}$	0.005

**Table 5:** Accelerometer Specification.

Parameter	Unit	Value
Measurement range	g	±15
Bandwidth	Hz	300
Initial bias	$\mathrm {\mu g}$	$<50$
Bias instability	$\mathrm {\mu g}$	7
Initial scaling error	ppm	340
Scale factor stability	ppm	150
Non-linearity	ppm	150
Misalignment error	deg	$<0.001$
Noise density	${\mathrm{\mu g/}}\sqrt{{\textrm{Hz}}}$	20
Velocity random walk	${\mathrm{mm/sec/}}\sqrt{{\textrm{hr}}}$	23

Measurements were acquired at a 100 Hz interval. In an iterative process, the longest allowable run time for the second phase, denoted as $t_{max}$, was determined to be 0.05 s through trial and error.

Data were recorded while the device was positioned on a three-axis calibration turn table. During the experiment, the true IMU biases can be easily computed according to the earth’s angular velocity, which is transformed into the turn table coordinate system.

**Figure 8:**
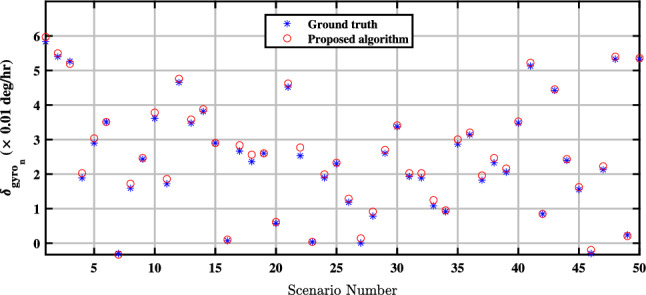
Comparison of estimated biases of the north gyroscope with exact values.

**Figure 9:**
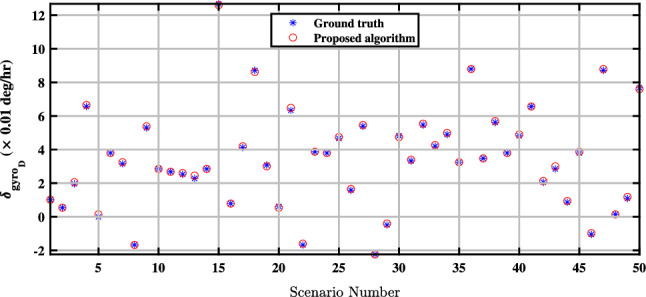
Comparison of estimated biases of the down gyroscope with exact values.

Figures 8 and 9 illustrate a comparison between the estimated biases of the north and down gyros with the exact values in 50 different scenarios. Evidently, the proposed algorithm accurately estimates the biases of both the north and down gyroscopes in real situations. However, due to the unobservability of the east gyroscope bias, the proposed algorithm cannot estimate it.

## Conclusion

This study introduces an innovative approach for SINS alignment, distinctively avoiding dependence on recursive estimation techniques like Kalman-based filtering. Recursive algorithms, particularly susceptible to slow convergence in azimuth angle error estimation, are bypassed. Instead, the methodology employs analytical relationships to calculate bias errors, resulting in a swifter estimation process. Furthermore, the accuracy of the proposed method is similar to that of the traditional fine alignment methods.

Simulation and experimental studies consistently validate the proposed approach’s superior convergence speed and equivalent accuracy compared to traditional fine alignment algorithms. In contrast to traditional methods, which demand over 10 min for azimuth angle estimation in stationary conditions^24^, the proposed approach achieves precise calculations of azimuth angle and gyroscope sensor biases within a mere 20 s, matching or surpassing the maximum accuracy achieved by traditional fine alignments.

A disadvantage of the proposed algorithm is that it is not applicable for a swaying or in-motion INS alignment, and its application is limited to stationary alignment and bias estimation. In addition, the first phase of the algorithm consists of a direct gyro-compassing algorithm as a coarse alignment, which provides the second phase with a rough estimation of attitude. According to the literature, to enable north-finding, a gyroscope needs a bias instability lower than the earth’s rotation rate^43^. Hence, the proposed algorithm requires a high-grade IMU that can measure earth’s rotation rate.

### Supplementary Information

Supplementary Information 1.

Supplementary Information 2.

## Data Availability

The datasets used and/or analysed during the current study available from the corresponding author on reasonable request.

## List of symbols

${[.]^{\textrm{B}}}$: Expressed in the body frame
${[.]^{\textrm{N}}}$: Expressed in the navigation frame
$\lambda ,\,\ell ,\,h$: Latitude, longitude, and height
${[{{\mathbf{\omega }}^{{\textrm{EI}}}}]^{\textrm{N}}}$: Angular velocity of Earth frame with respect to inertial frame and expressed in the navigation frame
${[{{\mathbf{\tilde{\omega }}}^{{\textrm{BI}}}}]^{\textrm{B}}}$: Measured angular velocity of body frame with respect to inertial frame and expressed in the body frame
$\omega _{x} ,\;\omega _{y} ,\;\omega _{z}$: Gyros output in the *x*, *y*, and *z* channels of the body frame
$\delta \omega _{x} ,\;\delta \omega _{y} ,\;\delta \omega _{z}$: Gyros error of in the x, y, and z channels of the body frame
$\varphi ,\,\theta ,\,\psi$: Roll, pitch, and yaw angles
${[{\textrm{T}}]^{{\textrm{BN}}}}$: Transformation matrix of the navigation frame to the body frame
$${[{{{\hat{\textrm{T}}}}}]^{{\textrm{BN}}}}$$: Estimation of $${[{\textrm{T}}]^{{\textrm{BN}}}}$$
${\textbf{q}}$: True quaternion vector
${{\textbf{q}}^{ - 1}}$: Inverse of true quaternion vector
${{\tilde{\textbf{q}}}}$: Error quaternion vector
${{\hat{\textbf{q}}}}$: Estimated quaternion vector
