# Supplementary material for: Analytical quaternion-based bias estimation algorithm for fast and accurate stationary gyro-compassing
Source: Sci Rep. 2024 Jul 9;14:15792. doi: 10.1038/s41598-024-66282-9 (PMC11233740; doi:10.1038/s41598-024-66282-9)
Supplement: Supplementary file 1 — Supplementary Information 1. [file 41598_2024_66282_MOESM1_ESM.pdf]

# Appendix A: Fine Alignment Accuracy

Consider the navigation error equations in the stationary condition [1]:

$$\begin{aligned}
&\varepsilon\dot{v}_{\mathrm{n}} = \mathrm{b_n} + w_{\mathrm{n}}^{\mathrm{a}} + 2\omega_{\mathrm{d}}\varepsilon v_{\mathrm{e}} + \mathrm{g}\varepsilon\theta \\
&\varepsilon\dot{v}_{\mathrm{e}} = \mathrm{b_e} + w_{\mathrm{e}}^{\mathrm{a}} - 2\omega_{\mathrm{d}}\varepsilon v_{\mathrm{n}} - \mathrm{g}\varepsilon\varphi \\
&\varepsilon\dot{v}_{\mathrm{d}} = \mathrm{b_d} + w_{\mathrm{d}}^{\mathrm{a}} - 2\omega_{\mathrm{n}}\varepsilon v_{\mathrm{e}} \\
&\varepsilon\dot{\varphi} = -\mathrm{d_n} - w_{\mathrm{n}}^{\mathrm{g}} + \tfrac{1}{\mathrm{R_e}}\varepsilon v_{\mathrm{e}} + \omega_{\mathrm{d}}\varepsilon\theta \\
&\varepsilon\dot{\theta} = -\mathrm{d_e} - w_{\mathrm{n}}^{\mathrm{g}} - \tfrac{1}{\mathrm{R_e}}\varepsilon v_{\mathrm{n}} - \omega_{\mathrm{d}}\varepsilon\varphi + \omega_{\mathrm{n}}\varepsilon\psi \\
&\varepsilon\dot{\psi} = -\mathrm{d_d} - w_{\mathrm{n}}^{\mathrm{g}} - \tfrac{\tan\lambda}{\mathrm{R_e}}\varepsilon v_{\mathrm{e}} - \omega_{\mathrm{n}}\varepsilon\theta \\
&\dot{\mathrm{b}}_{\mathrm{n}} = \dot{\mathrm{b}}_{\mathrm{e}} = \dot{\mathrm{b}}_{\mathrm{d}} = \dot{\mathrm{d}}_{\mathrm{n}} = \dot{\mathrm{d}}_{\mathrm{e}} = \dot{\mathrm{d}}_{\mathrm{d}} = 0
\end{aligned} \qquad \text{(A-1)}$$

where $\lambda$, $v_{\mathrm{n}}$, $v_{\mathrm{e}}$ and $v_{\mathrm{d}}$ denote latitude and north, east, and down terrestrial velocities, respectively. In the following, the roll, pitch, and yaw errors between the true and computed navigation frames are defined by $\varepsilon\varphi$, $\varepsilon\theta$ and $\varepsilon\psi$. Also, $\mathrm{d_i}$ and $\mathrm{b_i}$ (i=n,e,d) denote the components of gyros and accelerometer bias along the axes of the navigation frame.

In the stationary condition, the zero-velocity update (ZUPT) algorithm is considered, and thus the measurement vector is as follows:

$$\tilde{\mathbf{y}} = [\,\tilde{v}_{\mathrm{n}}\ \ \tilde{v}_{\mathrm{e}}\ \ \tilde{v}_{\mathrm{d}}\,]^{\mathrm{T}} = [\,0\ \ 0\ \ 0\,]^{\mathrm{T}} \qquad \text{(A-2)}$$

Performing the observability analysis to the ZUPT algorithm, as done in [1], it is understood that $\mathrm{b_n}$, $\mathrm{b_e}$ and $\mathrm{d_e}$ are unobservable states. Unobservable states cannot be estimated; thus, the accuracy of attitude estimation is restricted to the reference.

$$\begin{aligned}
&\min\{\varepsilon\varphi\} = \tfrac{\mathrm{b_e}}{\mathrm{g}} \\
&\min\{\varepsilon\theta\} = \tfrac{-\mathrm{b_n}}{\mathrm{g}} \\
&\min\{\varepsilon\psi\} = \tfrac{\omega_{\mathrm{d}}\mathrm{b_e}}{\mathrm{g}\omega_{\mathrm{n}}} + \tfrac{\mathrm{d_e}}{\omega_{\mathrm{n}}}
\end{aligned} \qquad \text{(A-3)}$$

# Appendix B: Alignment Error Analysis

In this appendix, the error analysis of the proposed alignment algorithm is presented. Consider an IMU in the stationary condition, accelerometers measure the surface reaction force, and gyroscopes measure the Earth's angular velocity, both in the body frame:

$$[\tilde{\mathbf{f}}]^{\mathrm{B}} = [-\mathbf{g}]^{\mathrm{B}} = \begin{bmatrix} \tilde{f}_{\mathrm{x}} \\ \tilde{f}_{\mathrm{y}} \\ \tilde{f}_{\mathrm{z}} \end{bmatrix} \quad , \quad [\tilde{\omega}^{\mathrm{BI}}]^{\mathrm{B}} = [\omega^{\mathrm{EI}}]^{\mathrm{B}} = \begin{bmatrix} \tilde{\omega}_{\mathrm{x}} \\ \tilde{\omega}_{\mathrm{y}} \\ \tilde{\omega}_{\mathrm{z}} \end{bmatrix} \tag{B-1}$$

The sign $\sim$ indicates the measurement of the desired quantity. In stationary condition, we also have:

$$[-\mathbf{g}]^{\mathrm{B}} = [\mathrm{T}]^{\mathrm{BN}}[-\mathbf{g}]^{\mathrm{N}} \quad , \quad [\omega^{\mathrm{EI}}]^{\mathrm{B}} = [\mathrm{T}]^{\mathrm{BN}}[\omega^{\mathrm{EI}}]^{\mathrm{N}} \tag{B-2}$$

Considering equation B-4, the following matrix can be formed:

$$\Big[[-\mathbf{g}]^{\mathrm{B}} \quad [\omega^{\mathrm{EI}}]^{\mathrm{B}} \quad [-\mathbf{g}]^{\mathrm{B}} \times [\omega^{\mathrm{EI}}]^{\mathrm{B}}\Big] = [\mathrm{T}]^{\mathrm{BN}} \Big[[-\mathbf{g}]^{\mathrm{N}} \quad [\omega^{\mathrm{EI}}]^{\mathrm{N}} \quad [-\mathbf{g}]^{\mathrm{N}} \times [\omega^{\mathrm{EI}}]^{\mathrm{N}}\Big] \tag{B-3}$$

Now consider $\mathbf{M} = \Big[[-\mathbf{g}]^{\mathrm{N}} \quad [\omega^{\mathrm{EI}}]^{\mathrm{N}} \quad [-\mathbf{g}]^{\mathrm{N}} \times [\omega^{\mathrm{EI}}]^{\mathrm{N}}\Big]$ , we would have:

$$[\mathrm{T}]^{\mathrm{BN}} = \Big[[-\mathbf{g}]^{\mathrm{B}} \quad [\omega^{\mathrm{EI}}]^{\mathrm{B}} \quad [-\mathbf{g}]^{\mathrm{B}} \times [\omega^{\mathrm{EI}}]^{\mathrm{B}}\Big] \ \mathbf{M}^{-1} \tag{B-4}$$

By solving equation B-4, an initial coarse estimate of the system attitude, $[\mathrm{T}]^{\mathrm{BN}}$ , is obtained as follows:

$$[\mathrm{T}]^{\mathrm{BN}} \approx \begin{bmatrix} \frac{\sec\lambda}{\omega^{\mathrm{EI}}}\tilde{\omega}_{\mathrm{x}} - \frac{\tan\lambda}{\mathrm{g}}\tilde{f}_{\mathrm{x}} & \frac{\sec\lambda}{\mathrm{g}\omega^{\mathrm{EI}}}(\tilde{f}_{\mathrm{z}}\tilde{\omega}_{\mathrm{y}} - \tilde{f}_{\mathrm{y}}\tilde{\omega}_{\mathrm{z}}) & \frac{-1}{\mathrm{g}}\tilde{f}_{\mathrm{x}} \\ \frac{\sec\lambda}{\omega^{\mathrm{EI}}}\tilde{\omega}_{\mathrm{y}} - \frac{\tan\lambda}{\mathrm{g}}\tilde{f}_{\mathrm{y}} & \frac{\sec\lambda}{\mathrm{g}\omega^{\mathrm{EI}}}(\tilde{f}_{\mathrm{x}}\tilde{\omega}_{\mathrm{z}} - \tilde{f}_{\mathrm{z}}\tilde{\omega}_{\mathrm{x}}) & \frac{-1}{\mathrm{g}}\tilde{f}_{\mathrm{y}} \\ \frac{\sec\lambda}{\omega^{\mathrm{EI}}}\tilde{\omega}_{\mathrm{z}} - \frac{\tan\lambda}{\mathrm{g}}\tilde{f}_{\mathrm{z}} & \frac{\sec\lambda}{\mathrm{g}\omega^{\mathrm{EI}}}(\tilde{f}_{\mathrm{y}}\tilde{\omega}_{\mathrm{x}} - \tilde{f}_{\mathrm{x}}\tilde{\omega}_{\mathrm{y}}) & \frac{-1}{\mathrm{g}}\tilde{f}_{\mathrm{z}} \end{bmatrix} \tag{B-5}$$

If the output of the sensors had no error, equation B-5 would also have no error and consequently the roll, pitch and yaw angles would be calculated exactly. In the following, the effect of the sensors error in the calculation of attitude matrix and Euler's angles is investigated. According to relations B-1, B-2 and B-4, we will have:

$$[\mathrm{T}]^{\mathrm{B}\hat{\mathrm{N}}} = \Big[[\tilde{\mathbf{f}}]^{\mathrm{B}} \quad [\tilde{\omega}^{\mathrm{BI}}]^{\mathrm{B}} \quad [\tilde{\mathbf{f}}]^{\mathrm{B}} \times [\tilde{\omega}^{\mathrm{BI}}]^{\mathrm{B}}\Big] \ \mathbf{M}^{-1} \tag{B-6}$$

In equation B-6, $[\mathbf{T}]^{\mathrm{B}\hat{\mathrm{N}}}$ , $[\tilde{\mathbf{f}}]^{\mathrm{B}}$ and $[\tilde{\omega}^{\mathrm{BI}}]^{\mathrm{B}}$ are respectively the estimation of the transfer matrix, the specific force and the angular rate sensed by the inertial sensors and are modeled as follows:

$$\begin{aligned} &\tilde{\mathbf{f}} = \mathbf{f} + \delta\mathbf{f} = -\mathbf{g}_{\mathrm{L}} + \delta\mathbf{f} \rightarrow [\tilde{\mathbf{f}}]^{\mathrm{B}} = [\delta\mathbf{f}]^{\mathrm{B}} - [\mathbf{g}]^{\mathrm{B}} \\ &\tilde{\omega}^{\mathrm{BI}} = \omega^{\mathrm{BI}} + \delta\omega^{\mathrm{BI}} = \omega^{\mathrm{EI}} + \delta\omega^{\mathrm{BI}} \rightarrow [\tilde{\omega}^{\mathrm{BI}}]^{\mathrm{B}} = [\omega^{\mathrm{EI}}]^{\mathrm{B}} + [\delta\omega^{\mathrm{BI}}]^{\mathrm{B}} \\ &[\mathrm{T}]^{\mathrm{B}\hat{\mathrm{N}}} = [\mathrm{T}]^{\mathrm{BN}}[\mathrm{T}]^{\mathrm{N}\hat{\mathrm{N}}} = [\mathrm{T}]^{\mathrm{BN}}[\bar{\mathrm{T}}]^{\hat{\mathrm{N}}\mathrm{N}} = [\mathrm{T}]^{\mathrm{BN}}[\mathbf{R}^{\hat{\mathrm{N}}\mathrm{N}}]^{\mathrm{N}} = [\mathrm{T}]^{\mathrm{BN}}\Big([\mathbf{E}]^{\mathrm{N}} + [\varepsilon\mathbf{R}^{\hat{\mathrm{N}}\mathrm{N}}]^{\mathrm{N}}\Big) \end{aligned} \tag{B-7}$$

In equation B-7, $\varepsilon\mathbf{R}^{\hat{\mathrm{N}}\mathrm{N}}$ is the skew-symmetric form of the tilt tensor ($\varepsilon\mathbf{r}^{\hat{\mathrm{N}}\mathrm{N}}$), which shows the tilt of the calculated transfer matrix ($[\mathrm{T}]^{\hat{\mathrm{N}}\mathrm{B}}$) from its real value ($[\mathrm{T}]^{\mathrm{NB}}$). According to equation B-7, we would have:

$$
\begin{aligned}
&[\mathrm{T}]^{\mathrm{BN}}\left([\mathbf{E}]^{\mathrm{N}}+[\varepsilon\mathbf{R}^{\hat{\mathrm{N}}\mathrm{N}}]^{\mathrm{N}}\right)= \\
&\left[[\delta\mathbf{f}]^{\mathrm{B}}-[\mathbf{g}]^{\mathrm{B}} \quad [\delta\omega^{\mathrm{BI}}]^{\mathrm{B}}+[\omega^{\mathrm{EI}}]^{\mathrm{B}} \quad \left([\delta\mathbf{f}]^{\mathrm{B}}-[\mathbf{g}]^{\mathrm{B}}\right)\times\left([\delta\omega^{\mathrm{BI}}]^{\mathrm{B}}+[\omega^{\mathrm{EI}}]^{\mathrm{B}}\right)\right]\mathbf{M}^{-1} \\
&\approx\left[[\delta\mathbf{f}]^{\mathrm{B}} \quad [\delta\omega^{\mathrm{BI}}]^{\mathrm{B}} \quad [\delta\mathbf{f}]^{\mathrm{B}}\times[\omega^{\mathrm{EI}}]^{\mathrm{B}}-[\mathbf{g}]^{\mathrm{B}}\times[\delta\omega^{\mathrm{BI}}]^{\mathrm{B}}\right]\mathbf{M}^{-1} \\
&+\left[-[\mathbf{g}]^{\mathrm{B}} \quad [\omega^{\mathrm{EI}}]^{\mathrm{B}} \quad -[\mathbf{g}]^{\mathrm{B}}\times[\omega^{\mathrm{EI}}]^{\mathrm{B}}\right]\mathbf{M}^{-1}
\end{aligned}
\tag{B-8}
$$

Subtracting equation B-4 from equation B-8, will result in:

$$
[\mathrm{T}]^{\mathrm{BN}}[\varepsilon\mathbf{R}^{\hat{\mathrm{N}}\mathrm{N}}]^{\mathrm{N}}=\left[[\delta\mathbf{f}]^{\mathrm{B}} \quad [\delta\omega^{\mathrm{BI}}]^{\mathrm{B}} \quad [\delta\mathbf{f}]^{\mathrm{B}}\times[\omega^{\mathrm{EI}}]^{\mathrm{B}}-[\mathbf{g}]^{\mathrm{B}}\times[\delta\omega^{\mathrm{BI}}]^{\mathrm{B}}\right]\mathbf{M}^{-1} \tag{B-9}
$$

Now consider the following definitions:

$$
[\delta\mathbf{f}]^{\mathrm{N}}=\begin{bmatrix}\delta f_{\mathrm{n}}\\ \delta f_{\mathrm{e}}\\ \delta f_{\mathrm{d}}\end{bmatrix} \quad , \quad [\delta\omega^{\mathrm{BI}}]^{\mathrm{N}}=\begin{bmatrix}\delta\omega_{\mathrm{n}}\\ \delta\omega_{\mathrm{e}}\\ \delta\omega_{\mathrm{d}}\end{bmatrix} \tag{B-10}
$$

Substituting equations B-10 into equation B-9, we would have:

$$
[\varepsilon\mathbf{R}^{\hat{\mathrm{N}}\mathrm{N}}]^{\mathrm{N}}=\begin{bmatrix}
+\frac{\sec\lambda}{\omega^{\mathrm{EI}}}\delta\omega_{\mathrm{d}} & -\frac{\sec\lambda}{\omega^{\mathrm{EI}}}\delta\omega_{\mathrm{e}}+\frac{\tan\lambda}{\mathrm{g}}\delta f_{\mathrm{e}} & -\frac{1}{\mathrm{g}}\delta f_{\mathrm{n}}\\
-\frac{\tan\lambda}{\mathrm{g}}\delta f_{\mathrm{e}}+\frac{\sec\lambda}{\omega^{\mathrm{EI}}}\delta\omega_{\mathrm{e}} & +\frac{\sec\lambda}{\omega^{\mathrm{EI}}}\delta\omega_{\mathrm{n}}-\frac{1}{\mathrm{g}}\delta f_{\mathrm{d}} & -\frac{1}{\mathrm{g}}\delta f_{\mathrm{e}}\\
+\frac{1}{\mathrm{g}}\delta f_{\mathrm{n}} & +\frac{1}{\mathrm{g}}\delta f_{\mathrm{e}} & -\frac{1}{\mathrm{g}}\delta f_{\mathrm{d}}
\end{bmatrix} \tag{B-11}
$$

Equation B-11 shows that in the presence of sensors error, $[\varepsilon\mathbf{R}^{\hat{\mathrm{N}}\mathrm{N}}]^{\mathrm{N}}$ is not necessarily skew-symmetric; But ideally, we should have:

$$
[\varepsilon\mathbf{R}^{\hat{\mathrm{N}}\mathrm{N}}]^{\mathrm{N}}=\begin{bmatrix}0 & -\varepsilon\psi & \varepsilon\theta\\ \varepsilon\psi & 0 & -\varepsilon\varphi\\ -\varepsilon\theta & \varepsilon\varphi & 0\end{bmatrix} \tag{B-12}
$$

According to the results of the proposed algorithm, it is seen that $\delta f_{\mathrm{d}}$, $\delta\omega_{\mathrm{n}}$ and $\delta\omega_{\mathrm{d}}$ are observable and $\delta f_{\mathrm{n}}$, $\delta f_{\mathrm{e}}$ and $\delta\omega_{\mathrm{e}}$ are unobservable states; It should be mentioned that the same result is obtained by performing the observability analysis to the fine alignment algorithm. Observable states are computed and compensated; thus, we would have:

$$
\delta f_{\mathrm{d}}=0 \quad , \quad \delta\omega_{\mathrm{n}}=0 \quad , \quad \delta\omega_{\mathrm{d}}=0 \tag{B-13}
$$

Applying equation B-13 to equation B-11, results in:

$$[\varepsilon\mathbf{R}^{\hat{\mathrm{N}}\mathrm{N}}]^{\mathrm{N}} = \begin{bmatrix} 0 & -\frac{\sec\lambda}{\omega^{\mathrm{EI}}}\delta\omega_{\mathrm{e}} + \frac{\tan\lambda}{\mathrm{g}}\delta f_{\mathrm{e}} & -\frac{1}{\mathrm{g}}\delta f_{\mathrm{n}} \\ +\frac{\sec\lambda}{\omega^{\mathrm{EI}}}\delta\omega_{\mathrm{e}} - \frac{\tan\lambda}{\mathrm{g}}\delta f_{\mathrm{e}} & 0 & -\frac{1}{\mathrm{g}}\delta f_{\mathrm{e}} \\ +\frac{1}{\mathrm{g}}\delta f_{\mathrm{n}} & +\frac{1}{\mathrm{g}}\delta f_{\mathrm{e}} & 0 \end{bmatrix} \tag{B-14}$$

Comparing equation B-14 with equation B-12, the error of the proposed algorithm is obtained:

$$\min(\varepsilon\varphi) = \frac{1}{\mathrm{g}}\delta f_{\mathrm{e}}, \min(\varepsilon\theta) = -\frac{1}{\mathrm{g}}\delta f_{\mathrm{n}}, \min(\varepsilon\psi) = +\frac{\sec\lambda}{\omega^{\mathrm{EI}}}\delta\omega_{\mathrm{e}} - \frac{\tan\lambda}{\mathrm{g}}\delta f_{\mathrm{e}} \tag{B-15}$$

The results obtained in equation B-15 are in full compliance with the error analysis of the fine alignment algorithm given in [1]."

# References


[1] Bar-Itzhack, I.Y., Berman, N.: Control theoretic approach to inertial navigation systems. Journal of Guidance, control, and Dynamics **11**(3), 237–245 (1988)
