# Supplementary material for: Analytical quaternion-based bias estimation algorithm for fast and accurate stationary gyro-compassing
Source: Sci Rep. 2024 Jul 9;14:15792. doi: 10.1038/s41598-024-66282-9 (PMC11233740; doi:10.1038/s41598-024-66282-9)
Supplement: Supplementary file 2 — Supplementary Information 2. [file 41598_2024_66282_MOESM2_ESM.doc]

clear
close all
clc
format compact
format long g
DATA=[];
%==========================================================================
D2R=pi/180;
R2D=180/pi;
%=========================
Re=6378137;
w_ei=7292115e-11;
kmax=200;
WB=waitbar(0,'Please wait ...');
for k=1:kmax
 %=========================
 lat=random('unif',-85,85)*D2R;
 lon=random('unif',5,355)*D2R;
 h=random('unif',0,5000);
 g=gravitywgs84(h,lat*R2D);
 ST=.01;
 %==========================================================================
 ba=100*1E-6*g; %100 micro g
 BA=random('unif',-1,1,3,1)*ba;
 ASE=20/1E6; %Accelerometer Scale-factor Error, 50 ppm
 AME=5*D2R/3600; %Accelerometer Misalignment Error, 10 ArcSeconds
 ASME=eye(3)+diag(random('unif',-1,1,3,1))*ASE+[0 random('unif',-1,1,1,2); random('unif',-1,1) 0 random('unif',-1,1); random('unif',-1,1,1,2) 0]*AME;
 VRW=0.05/60;
 %==========================================================================
 dg=.01*D2R/3600; %0.01 deg/hr
 DG=random('unif',-1,1,3,1)*dg;
 GSE=50/1E6; %Gyro Scale-factor Error, 20 ppm
 GME=10*D2R/3600; %Gyro Misalignment Error, 5 ArcSeconds
 GSME=eye(3)+diag(random('unif',-1,1,3,1))*GSE+[0 random('unif',-1,1,1,2); random('unif',-1,1) 0 random('unif',-1,1); random('unif',-1,1,1,2) 0]*GME;
 ARW=0.01*D2R/60;
 %==========================================================================
 vn=0;
 ve=0;
 vd=0;
 %=============================
 psi=random('unif',-180,180)*D2R;
 theta=random('unif',-90,90)*D2R;
 phi=random('unif',-180,180)*D2R;
 T_BN=angle2dcm(psi,theta,phi);
 %==========================================================================
 wn=w_ei*cos(lat);
 wd=-w_ei*sin(lat);
 w_EI_N=[wn;0;wd];
 w_BI_B=T_BN*w_EI_N;
 %==================
 g_N=[0;0;g];
 a_BI_B=T_BN*(-g_N);
 %==========================================================================
 TF=20;
 w_BI_Ba=0;
 a_BI_Ba=0;
 for i=ST:ST:TF
 w_BI_Bm=GSME*w_BI_B+DG+random('Normal',0,ARW,3,1);
 a_BI_Bm=ASME*a_BI_B+BA+random('Normal',0,VRW,3,1);
 w_BI_Ba=w_BI_Ba+w_BI_Bm;
 a_BI_Ba=a_BI_Ba+a_BI_Bm;
 end
 w_BI_Ba=w_BI_Ba/TF*ST;
 a_BI_Ba=a_BI_Ba/TF*ST;
 fx=a_BI_Ba(1);
 fy=a_BI_Ba(2);
 fz=a_BI_Ba(3);
 wx=w_BI_Ba(1);
 wy=w_BI_Ba(2);
 wz=w_BI_Ba(3);
 %==========================================================================
 T_BNc=[sec(lat)/w_ei*wx-tan(lat)/g*fx, sec(lat)/(g*w_ei)*(fz*wy-fy*wz), -fx/g;...
 sec(lat)/w_ei*wy-tan(lat)/g*fy, sec(lat)/(g*w_ei)*(fx*wz-fz*wx), -fy/g;...
 sec(lat)/w_ei*wz-tan(lat)/g*fz, sec(lat)/(g*w_ei)*(fy*wx-fx*wy), -fz/g];
 %T_BNc=1.5*T_BNc-0.5*T_BNc*T_BNc.'*T_BNc;
 [psic,thetac,phic]=dcm2angle(T_BNc);
 %==========================================================================
 ST=.01;
 TF=.01;
 psi0=psic;
 theta0=thetac;
 phi0=phic;
 T_BN0=angle2dcm(psi0,theta0,phi0);
 Ipsi=0;
 Itheta=0;
 Iphi=0;
 T_BNt=T_BN0;
 %======================================================================
 v_B_E_Bt=[vn; ve; vd];
 Ivn=0;
 Ive=0;
 Ivd=0;
 %======================================================================
 Iepsi=0;
 Ietheta=0;
 Iephi=0;
 Iephietheta=0;
 Iepsietheta=0;
 Iepsiephi=0;
 Iephiethetaepsi=0;
 %======================================================================
 for i=ST:ST:TF
 %==============================================================
 w_BN_B=w_BI_Ba-T_BN0*w_EI_N;
 s=-w_BN_B*ST;
 S=[0 -s(3) +s(2); +s(3) 0 -s(1); -s(2) +s(1) 0];
 norm_s=norm(s);
 exp_s=eye(3)+sin(norm_s)/norm_s*S+(1-cos(norm_s))/norm_s^2*S^2;
 T_BNt=exp_s*T_BNt;
 T_BNt=1.5*T_BNt-0.5*(T_BNt*T_BNt.')*T_BNt;
 %==============================================================
 [psit,thetat,phit]=dcm2angle(T_BNt);
 Ipsi=Ipsi+psit*ST;
 Itheta=Itheta+thetat*ST;
 Iphi=Iphi+phit*ST;
 %==============================================================
 %d/dt(v_B_E_Bt)=T_BN0.'*a_BI_Ba+g_N-2*cross(w_EI_N,v_B_E_Bt);
 v_B_E_Bt=v_B_E_Bt+(T_BN0.'*a_BI_Ba+g_N)*ST;
 vnt=v_B_E_Bt(1);
 vet=v_B_E_Bt(2);
 vdt=v_B_E_Bt(3);
 Ivn=Ivn+vnt*ST;
 Ive=Ive+vet*ST;
 Ivd=Ivd+vdt*ST;
 %==============================================================
 Ietheta=Ietheta+(thetat-theta0)*ST;
 Iephi=Iephi+(phit-phi0)*ST;
 Iephietheta=Iephietheta+(phit-phi0)*(thetat-theta0)*ST;
 Iepsietheta=Iepsietheta+(psit-psi0)*(thetat-theta0)*ST;
 Iepsiephi=Iepsiephi+(psit-psi0)*(phit-phi0)*ST;
 Iephiethetaepsi=Iephiethetaepsi+(phit-phi0)*(thetat-theta0)*(psit-psi0)*ST;
 %==============================================================
 end
 %==========================================================================
 dfn=(vnt-2*wd*Ive)/TF;
 dfe=(vet+2*wd*Ivn-2*wn*Ivd)/TF;
 dfd=(vdt+2*wn*Ive)/TF;
 %==========================================================================
 dwn=(+1/Re*Ive...
 -wd*cos(psi0)*Ietheta...
 -wd*cos(theta0)*sin(psi0)*Iephi...
 -wd*sin(theta0)*sin(psi0)*Iephietheta...
 -sin(psi0)*(thetat-theta0)...
 +cos(theta0)*cos(psi0)*(phit-phi0)...
 +cos(theta0)*sin(psi0)*(phit-phi0)*(psit-psi0)...
 +cos(psi0)*(psit-psi0)*(thetat-theta0))/TF;
 dwe=(-1/Re*Ivn...
 +(wd*cos(psi0)*cos(theta0)+wn*sin(theta0))*Iephi...
 -wd*sin(psi0)*Ietheta-wn*Iepsi...
 +wd*cos(psi0)*Iepsietheta...
 +wd*cos(theta0)*sin(psi0)*Iepsiephi...
 -wn*cos(psi0)^2*cos(theta0)*Iephietheta...
 -wn*cos(psi0)*cos(theta0)*sin(psi0)*Iephiethetaepsi...
 +cos(psi0)*(thetat-theta0)+cos(theta0)*sin(psi0)*(phit-phi0)...
 +sin(psi0)*sin(theta0)*(phit-phi0)*(thetat-theta0))/TF;
 dwd=(-tan(lat)/Re*Ive...
 +wn*cos(psi0)*Ietheta...
 +wn*cos(theta0)*sin(psi0)*Iephi...
 +wn*sin(psi0)*sin(theta0)*Iephietheta...
 -sin(theta0)*(phit-phi0)+(psit-psi0)...
 +cos(psi0)^2*cos(theta0)*(phit-phi0)*(thetat-theta0)...
 +cos(psi0)*cos(theta0)*sin(psi0)*(phit-phi0)*(thetat-theta0)*(psit-psi0))/TF;
 %==========================================================================
 df=T_BN0*[dfn;dfe;dfd];
 fx=fx-df(1);
 fy=fy-df(2);
 fz=fz-df(3);
 %=======================
 dw=T_BN0*[dwn;dwe*0;dwd];
 wx=wx-dw(1);
 wy=wy-dw(2);
 wz=wz-dw(3);
 %=======================
 T_BNcm=[sec(lat)/w_ei*wx-tan(lat)/g*fx, sec(lat)/(g*w_ei)*(fz*wy-fy*wz), -fx/g;...
 sec(lat)/w_ei*wy-tan(lat)/g*fy, sec(lat)/(g*w_ei)*(fx*wz-fz*wx), -fy/g;...
 sec(lat)/w_ei*wz-tan(lat)/g*fz, sec(lat)/(g*w_ei)*(fy*wx-fx*wy), -fz/g];
 %T_BNc=1.5*T_BNc-0.5*T_BNc*T_BNc.'*T_BNc;
 [psicm,thetacm,phicm]=dcm2angle(T_BNcm);
 %==========================================================================
 DATA=[DATA; lat lon h phi theta psi reshape(ASME.',1,9) BA.' reshape(GSME.',1,9) DG.' psicm psic...
 [T_BN.'*a_BI_Ba-(-g_N)]' [dfn dfe dfd] [T_BN.'*w_BI_Ba-w_EI_N]' [dwn dwe dwd]];
 %==========================================================================
 waitbar(k/kmax)
end
close(WB)
%==========================================================================
MeanFine=mean(abs(DATA(:,40).*sec(DATA(:,1))))/w_ei*R2D
MeanDiff=mean(abs((DATA(:,31)-DATA(:,6)))*R2D)
%==========================================================================
plot(abs(DATA(:,40).*sec(DATA(:,1)))/w_ei*R2D,'b');hold on;
plot(abs((DATA(:,31)-DATA(:,6)))*R2D,'r-.');hold off;
legend(['Fine Alignment; Mean=' num2str(mean(abs(DATA(:,40).*sec(DATA(:,1))))/w_ei*R2D)],...
 ['Differential Alignment; Mean=' num2str(mean(abs((DATA(:,31)-DATA(:,6)))*R2D))])
xlabel('Scenario Number')
ylabel('\psi Angle Error (deg)')
grid
%==========================================================================
% figure
% subplot(3,1,1)
% plot(DATA(:,33)/(1E-6*g),'b*');hold on;plot(DATA(:,36)/(0.01*ba),'ro');hold off;
% ylabel('\delta_{acc._n} (\times \mug)')
% legend('True','Estimated')
% grid
% subplot(3,1,2)
% plot(DATA(:,34)/(1E-6*g),'b*');hold on;plot(DATA(:,37)/(0.01*ba),'ro');hold off;
% ylabel('\delta_{acc._e} (\times \mug)')
% grid
% subplot(3,1,3)
% plot(DATA(:,35)/(1E-6*g),'b*');hold on;plot(DATA(:,38)/(0.01*ba),'ro');hold off;
% xlabel('Scenario Number')
% ylabel('\delta_{acc._d} (\times \mug)')
% grid
%==========================================================================
figure
plot(DATA(:,39)/(.01*D2R/3600),'b*');hold on;plot(DATA(:,42)/(dg),'ro');hold off;
ylabel('\delta_{gyro_n} (\times 0.01 deg/hr)')
grid
figure
plot(DATA(:,40)/(.01*D2R/3600),'b*');hold on;plot(DATA(:,43)/dg,'ro');hold off;
ylabel('\delta_{gyro_e} (\times 0.01 deg/hr)')
grid
legend('True','Estimated')
figure
plot(DATA(:,41)/(.01*D2R/3600),'b*');hold on;plot(DATA(:,44)/dg,'ro');hold off;
xlabel('Scenario Number')
ylabel('\delta_{gyro_d} (\times 0.01 deg/hr)')
grid
%==========================================================================

[*Published with MATLAB® R2020a*](https://www.mathworks.com/products/matlab)
